# Hymenoptera Genome Database: new genomes and annotation datasets for improved go enrichment and orthologue analyses

**DOI:** 10.1093/nar/gkab1018

**Published:** 2021-11-08

**Authors:** Amy T Walsh, Deborah A Triant, Justin J Le Tourneau, Md Shamimuzzaman, Christine G Elsik

**Affiliations:** Division of Animal Sciences, University of Missouri, Columbia, MO 65211, USA; Division of Animal Sciences, University of Missouri, Columbia, MO 65211, USA; Division of Animal Sciences, University of Missouri, Columbia, MO 65211, USA; Division of Animal Sciences, University of Missouri, Columbia, MO 65211, USA; Division of Animal Sciences, University of Missouri, Columbia, MO 65211, USA; Division of Plant Science & Technology, University of Missouri, Columbia, MO 65211, USA; MU Institute for Data Science & Informatics, University of Missouri, Columbia, MO 65211, USA

## Abstract

We report an update of the Hymenoptera Genome Database (HGD; http://HymenopteraGenome.org), a genomic database of hymenopteran insect species. The number of species represented in HGD has nearly tripled, with fifty-eight hymenopteran species, including twenty bees, twenty-three ants, eleven wasps and four sawflies. With a reorganized website, HGD continues to provide the HymenopteraMine genomic data mining warehouse and JBrowse/Apollo genome browsers integrated with BLAST. We have computed Gene Ontology (GO) annotations for all species, greatly enhancing the GO annotation data gathered from UniProt with more than a ten-fold increase in the number of GO-annotated genes. We have also generated orthology datasets that encompass all HGD species and provide orthologue clusters for fourteen taxonomic groups. The new GO annotation and orthology data are available for searching in HymenopteraMine, and as bulk file downloads.

## INTRODUCTION AND OVERVIEW

The Hymenoptera Genome Database (HGD; http://HymenopteraGenome.org) (1) is a genome informatics resource for insects of the order Hymenoptera (bees, ants, wasps and sawflies). Widely accessible and efficient sequencing technologies have made it possible for researchers of hymenopteran insects to use genome sequencing to address a wide variety of questions. For example, the hymenopteran species exhibit a range of eusociality levels, from solitary to advanced eusocial lifestyles, and are used to investigate topics such as evolution of eusociality, molecular regulation of division of labor and epigenetics of behavior (2–8). Hymenopteran genome sequencing projects are also used to develop models for evolution and adaptation to fungal and plant symbioses (9–13), evolution of social parasitism (14), parasitoid biology (15–17), impact of endosymbionts (13,18,19), adaptation of invasive species (20), ecological speciation (21), transitions to asexual reproduction (21), phenotypic plasticity (8,14,22), selfish B chromosome drive (23) and the evolution of miniaturization (16). In addition to developing biological models, genome sequencing is used to address topics related to agriculture, such as response to pesticides (24) and roles as biological control agents (15,16). Furthermore, the Hymenoptera are the largest group of pollinators (25). The goal of HGD is to make the hymenopteran genome sequences and associated data easily accessible for further investigation.

As reported previously (1), HGD provides JBrowse (26) genome browsers with Apollo (27) annotation tools, integrated with a BLAST server (28,29), for visual inspection of genes in their genomic context. The primary method of searching HGD is with HymenopteraMine, a data mining warehouse for querying and exporting disparate sources of gene annotation data. HymenopteraMine, based on the InterMine data mining warehouse (30), integrates data from external sources, including RefSeq (31), UniProt (32), InterPro (33), OrthoDB (34), KEGG (35), PubMed (36) and BioGrid (37). Furthermore, by including the Dipteran outgroup, *Drosophila melanogaste*r, in HymenopteraMine, hymenopteran genes can be connected to *D. melanogaster* data in Reactome (38) and IntAct (39) via orthologous relationships. First reported in 2016 (1), HymenopteraMine provides several search tools, including a simple keyword search, the QueryBuilder for constructing custom queries, pre-constructed template query menus, the List Tool to upload lists of identifiers and the Regions Search tool to query for genome features based on a list of genomic coordinates. Report pages and query outputs are provided as tables that can be further modified by clicking icons in column headings and by using menus for managing columns, filters and relationships. Tables can be exported in several formats, including tab-delimited. Detailed methods for using the search tools have been previously published (40), and are available by clicking the ‘LEARNING’ tab in the navigation bar on the HGD home page. Here, we report a more than doubling of the number of hymenopteran species in HGD, as well as the generation of two new datasets, HGD-Ortho and HGD GO Annotation, which are available for searching in HymenopteraMine and available for bulk download.

## NEW AND UPDATED GENOMES

The current HGD release has a total of 58 hymenopteran genomes. Since the previous HGD update report (1), we have incorporated genomes of 38 additional species and have updated genomes and/or gene sets of 13 species (Table 1). The acquisition of new genomes expands the insect groups previously hosted in HGD, for example, increasing from 10 to 23 ant species and 9 to 20 bee species. Previously, *Nasonia vitripennis* was both the only wasp and the only parasitoid in HGD. Now HGD hosts eleven wasp species, nine of which are members of the Parasitoida infraorder, and two of which are social non-parasitoid species. HGD also now hosts genomes of four sawfly species, a group previously not represented at all in HGD. All of the genomes are supported with JBrowse/Apollo genome browsers, BLAST and HymenopteraMine.

**Table 1.** Genomes in HGD

Group	Family	Species	New/Updated^a^	Assembly accession^b^	Assembly name	Ref^c^
Ants	Formicidae	*Acromyrmex echinatior*		GCF_204515.1	Aech_3.9	(9)
		*Atta cephalotes*		GCF_143395.1	Attacep1.0	(10)
		*Atta colombica*	N	GCF_1594045.1	Acol1.0	(11)
		*Camponotus floridanus*	U	GCF_3227725.1	Cflo_v7.5	(6)
		*Cardiocondyla obscurior*			Cobs_1.4	(20)
		*Cyphomyrmex costatus*	N	GCF_1594065.1	Ccosl1.0	(11)
		*Dinoponera quadriceps*	N	GCF_1313825.1	ASM131382v1	(22)
		*Formica exsecta*	N	GCF_3651465.1	ASM365146v1	(18)
		*Harpegnathos saltator*	U	GCF_3227715.1	Hsal_v8.5	(6)
		*Linepithema humile*		GCF_217595.1	Lhum_UMD_V04	(41)
		*Monomorium pharaonis*	N	GCF_3260585.2	ASM326058v2	(42)
		*Nylanderia fulva*	N	GCF_5281655.1	TAMU_Nfulva_1	NP
		*Odontomachus brunneus*	N	GCF_10583005.1	Obru_v1	NP
		*Ooceraea biroi*	U	GCF_3672135.1	Obir_v5.4	(7)
		*Pogonomyrmex barbatus*	U	GCF_187915.1	Pbar_UMD_V03	(43)
		*Pseudomyrmex gracilis*	N	GCF_2006095.1	ASM200609v1	(12)
		*Solenopsis invicta*	U	GCF_188075.2	Si_gnH	(44)
		*Temnothorax curvispinosus*	N	GCF_3070985.1	ASM307098v1	NP
		*Trachymyrmex cornetzi*	N	GCF_1594075.1	Tcor1.0	(11)
		*Trachymyrmex septentrionalis*	N	GCF_1594115.1	Tsep1.0	(11)
		*Trachymyrmex zeteki*	N	GCF_1594055.1	Tzet1.0	(11)
		*Vollenhovia emeryi*	N	GCF_949405.1	V.emery_V1.0	(14)
		*Wasmannia auropunctata*		GCF_956235.1	wasmannia.A_1	NP
Bees	Apidae	*Apis cerana*	N	GCF_1442555.1	ACSNU-2.0	(45)
		*Apis dorsata*	N	GCF_469605.1	Apis_dorsata_1.3	(46)
		*Apis florea*	N	GCF_184785.2	Aflo_1.1	(46)
		*Apis mellifera*	U	GCF_3254395.2	Amel_HAv3.1	(47)
		*Bombus bifarius*	N	GCF_11952205.1	Bbif_JDL3187	(48)
		*Bombus impatiens*	U	GCF_188095.3	BIMP_2.2	(2)
		*Bombus terrestris*	U	GCF_214255.1	Bter_1	(2)
		*Bombus vancouverensis*	N	GCF_11952275.1	Bvanc_JDL1245	(48)
		*Bombus vosnesenskii*	N	GCF_11952255.1	Bvos_JDL3184-5_v1.1	(48)
		*Ceratina calcarata*	N	GCF_1652005.1	ASM165200v1	(3)
		*Eufriesea mexicana*	U	GCF_1483705.1	ASM148370v1	(4)
		*Habropoda laboriosa*	U	GCF_1263275.1	ASM126327v1	(4)
		*Melipona quadrifasciata*	U	GCA_1276565.1	ASM127656v1	(4)
	Halictidae	*Dufourea novaeangliae*	U	GCF_1272555.1	ASM127255v1	(4)
		*Lasioglossum albipes*			Lalb_v2	(5)
		*Megalopta genalis*	N	GCF_11865705.1	USU_MGEN_1.2	(8)
		*Nomia melanderi*	N	GCF_3710045.1	USU_Nmel_1.2	(49)
	Megachilidae	*Megachile rotundata*		GCF_220905.1	MROT_1	(4)
		*Osmia bicornis*	N	GCF_4153925.1	Obicornis_v3	(24)
		*Osmia lignaria*	N	GCF_12274295.1	USDA_Olig_1	NP
Sawflies	Cephidae	*Cephus cinctus*	N	GCF_341935.1	Ccin1	(50)
	Diprionidae	*Neodiprion lecontei*	N	GCF_1263575.1	Nlec1.0	(51)
	Orussidae	*Orussus abietinus*	N	GCF_612105.2	Oabi_2	(52)
	Tenthredinidae	*Athalia rosae*	N	GCF_344095.2	Aros_2	(52)
Wasps (non-parasitoid)	Vespidae	*Polistes canadensis*	N	GCF_1313835.1	ASM131383v1	(22)
		*Polistes dominula*	N	GCF_1465965.1	Pdom_r1.2	(53)
Wasps (parasitoid)	Agaonidae	*Ceratosolen solmsi*	N	GCF_503995.1	CerSol_1	(13)
	Braconidae	*Chelonus insularis*	N	GCF_13357705.1	ASM1335770v1	NP
		*Diachasma alloeum*	N	GCF_1412515.2	Dall2.0	(21)
		*Fopius arisanus*	N	GCF_806365.1	ASM80636v1	(15)
		*Microplitis demolitor*	N	GCF_572035.2	Mdem2	(19)
	Cynipidae	*Belonocnema treatae*	N	GCF_10883055.1	B_treatae_v1	NP
	Encyrtidae	*Copidosoma floridanum*	N	GCF_648655.2	Cflo_2	(54)
	Pteromalidae	*Nasonia vitripennis*	U	GCF_9193385.2	Nvit_psr_1.1	(23)
	Trichogrammatidae	*Trichogramma pretiosum*	N	GCF_599845.2	Tpre_2	(16)
Fly (Dipteran outgroup)	Drosophilidae	*Drosophila melanogaster*	N	GCF_1215.4	Release_6_plus_ISO1_MT	(55)

^a^New (N) genome or updated (U) genome assembly and/or gene set since the previous update report (1). A blank cell in the New/Updated column indicates no changes in genome assembly or gene set.

^b^A blank cell in the assembly accession column indicates the genome assembly is not available at NCBI.

^c^NP denotes ‘not published’. Links for data usage policies for these species are provided on the HGD ‘Genome Publications’ page.

## REVAMPED WEBSITE

To better organize the growing number of genomes in HGD, we have overhauled the website. HGD now combines all species into one unified website, rather than separating species into the old divisions for ‘BeeBase’, ‘NasoniaBase’ and ‘Ant Genomes Portal’. The older webpages are available in the ‘Archive’ tab on the navigation bar. The ‘Downloads’ tab in the HGD main navigation bar provides access to files for all species organized into data type. There are also new pages for Learning (with documentation and examples), Release Notes, Community Data, and Contributing Data.

## NEW GENE ONTOLOGY ANNOTATION DATA

For most of the HGD species, the number of genes with UniProt-GOA annotations is not sufficient for Gene Ontology (GO) enrichment analysis. The three species with the highest numbers of UniProt-GOA annotated genes are *Atta cephalotes* (7760 genes), *Apis mellifera* (4331 genes) and *Nasonia vitripennis* (3160 genes). Forty HGD species have fewer than 100 UniProt-GOA annotated genes. To perform GO enrichment analysis of these species with few annotations, researchers must annotate the genes themselves, or identify orthologues in a well-annotated species and perform GO enrichment based on a background gene list from that species. HymenopteraMine has always provided tools for easy GO enrichment analysis for the few UniProt-GOA annotated species. To make these tools available for all species we have enhanced the GO annotation data obtained from UniProt-GOA by generating GO annotation data for all species.

GO annotations were generated from combined sources: (i) UniProt-GOA (56), (ii) transfer of GO terms from InterPro matches (33), (iii) transfer of GO terms based on homology and InterPro domain content. GO annotations for each species, when available, were parsed from the goa_uniprot_all.gaf file (UniProt-GOA; UniProt Knowledgebase Release 2020_04, downloaded from ftp://ftp.ebi.ac.uk/pub/databases/GO/goa/UNIPROT/) and protein ids were converted to RefSeq gene ids using the UniProt idmapping_selected.tab.gz file (ftp://ftp.uniprot.org/pub/databases/uniprot/current_release/knowledgebase/idmapping/). UniProt-GOA annotations were not used if the annotated protein mapped to more than one gene. We supplemented GO annotations using computational methods. First, we used InterProScan (57) to identify protein domains from the SMART, SUPERFAMILY, Panther and Pfam databases (58–61) using the -goterms option to lookup GO terms for matching domains. Second, we used the FASTA (62) sequence comparison program to perform protein searches between each species and four annotated reference species. *D. melanogaster* and human were used as reference species because they have highly curated GO annotation datasets. *D. melanogaster* protein sequences and GO annotations were obtained from FlyBase (release FB2020_02) (63). Human proteins were obtained from UniProt, and human GO annotations from UniProt-GOA. *A. mellifera* and *A. cepahotes* were also used as reference species because they had the highest number of UniProt-GOA annotations among the hymenopteran species in HGD. GO terms were transferred to the query proteins from (i) reciprocal best-hit reference proteins and (ii) best-hit reference proteins that were not reciprocal, but had identical protein domain content identified by InterProScan. Molecular Function and Cellular Component terms, but not Biological Process Terms, were transferred from the human reference protein dataset. Inferred annotations were added using inter-ontology links (64) in the go.obo file downloaded from the Gene Ontology Consortium (release date 2020-08-11, doi:10.5281/zenodo.2529950; http://geneontology.org/docs/download-ontology/) (65,66). Finally, all parents of GO terms were added to annotations using the go.obo file.

**Figure 1.**
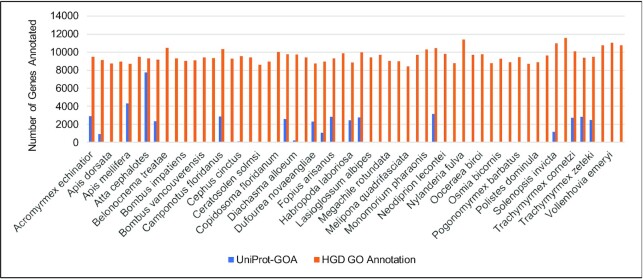
The number of genes with GO annotations for each species in the UniProt-GOA and HGD GO Annotation datasets.

The number of genes in HGD with GO annotations has significantly increased (Figure 1) due to adding GO annotations generated in our pipeline to the UniProt data. The total number of genes in HGD annotated with GO increased from 47 789 when UniProt was the sole source of GO annotation data to 553 866 after adding GO annotations that we computed based on sequence comparison and protein domain content, and the mean number of annotated genes per species increased from 824 to 9549. The annotations are available in HymenopteraMine, allowing for GO enrichment analysis, as described previously (40). Supplementary File 1 provides an example showing GO enrichment analysis using the HymenopteraMine List Tool with a list of *Bombus vosnesenskii* gene identifiers provided in Supplementary File 2. Another example with detailed instructions for GO enrichment using the List Tool is available by selecting ‘TUTORIAL EXAMPLES’ under the ‘LEARNING’ tab in the HGD navigation bar. In addition to HymenopteraMine, the GO annotations are available as downloadable files in Gene Annotation File (GAF) format (http://geneontology.org/docs/go-annotation-file-gaf-format-2.1/) and in a format that can be used with GeneMerge, a command-line GO enrichment software package (67).

## NEW ORTHOLOGUE DATA

HymenopteraMine has always included orthologue data from OrthoDB (34). However, OrthoDB contains only 36 of the 58 hymenopteran species currently in HGD. To provide orthologues for all species, we have generated new orthologue data using Orthologer (34), the same software developed and used to compute orthologues by OrthoDB. Our new orthologue dataset, called HGD-Ortho, was computed for 14 taxonomic groups based on the NCBI Taxonomy database (36), ranging from the level of genus to superorder (Table 2). When querying the data, users can select a taxonomic group representing the last common ancestor, thereby controlling evolutionary distance, which can affect the level of sequence divergence and number of paralogs within a cluster. Species lists for each taxonomic group are provided in Supplementary File 3. HGD-Ortho data are available for searching in HymenopteraMine, and as bulk downloadable files. HymenopteraMine still maintains the OrthoDB orthologue data so that researchers interested in the supported species can follow their HymenopteraMine work with other resources available at the OrthoDB website using orthologue cluster identifiers common to both resources.

**Table 2.** Taxonomic groups in the HGD-Ortho dataset

Taxonomic group^a^	Rank	Number of species
Holometabola	superorder	59
>Hymenoptera	order	58
>>Aculeata	infraorder	45
>>>Apoidea	superfamily	20
>>>>Apidae	family	13
>>>>>>Apis	genus	4
>>>>>>Bombus	genus	5
>>>>Halictidae	family	4
>>>Formicoidea^a^	superfamily	23
>>>>Formicidae^b^	family	23
>>>>>Formicinae	subfamily	4
>>>>>Myrmicinae	subfamily	14
>>Parasitoida	infraorder	9
>>>>Chalcidoidea	family	4
>>>>Ichneumonoidea	family	4

^a^Indentations shown as ‘>’ represent taxonomic hierarchy.

^b^In HGD, all species of the superfamily Formicoidea are within the family Formicidae, and are labeled as the latter in HymenopteraMine.

**Figure 2.**
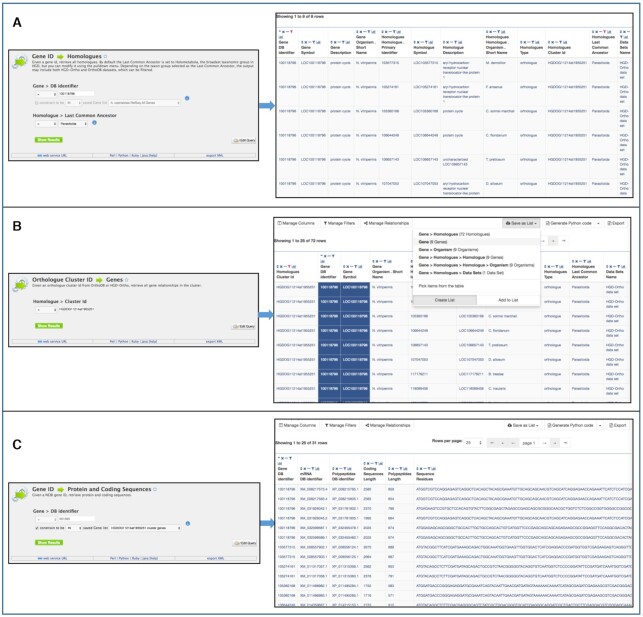
An example of the use of the HGD-Ortho dataset to gather orthologue sequences for later molecular evolution analyses. (**A**) ‘Gene ID → Homologues’ template query and output identifying orthologues among Parasitoida, along with the orthologue cluster identifier, for a gene of *Nasonia vitripennis*. The *N. vitripennis* RefSeq gene id is entered into the box, and ‘Parasitoida’ is selected in the pulldown menu. (**B**) ‘Orthologue Cluster ID → Genes’ template query and output, showing all pairwise gene relationships in the orthologue cluster identified the previous query. The ‘Save as List’ menu in the output page is used to save a list of genes in the orthologue cluster. (**C**) ‘Gene ID → Protein and Coding Sequences’ template query and output. Rather than entering a single ‘Gene DB Identifier’, the box next to ‘constrain to be IN’ is checked and the gene list saved previously is selected in the pulldown menu. The output includes coding sequences, protein sequences, identifiers and sequence lengths, which can then be used to select the longest coding sequence of genes with multiple transcripts for downstream molecular evolution analyses. The protein sequences are in the rightmost column and are not visible in this figure. The ‘Export’ button in the top right corner can be used to export the sequences.

To demonstrate the use of HGD-Ortho, we describe how to use HymenopteraMine to gather protein and coding sequences that can be used to investigate sequence evolution of the cycle gene in *Nasonia vitripennis* in comparison to other parasitoid species. This example involves saving a list of identifiers for use in template queries. While you can save a list temporarily without logging in to a MyMine account, saving a list while logged in stores the list in your account for future sessions. Account registration is freely available by clicking ‘Log In’ near the upper right corner of the HymenopteraMine home page. Use the ‘Gene ID → Homologues’ template query, found under the ‘Homology’ tab in the template category bar in the middle of the HymenopteraMine home page. Enter the RefSeq gene id (100118796) and select ‘Parasitoida’ as the ‘Last Common Ancestor’ to retrieve the orthologue cluster id (Figure 2A), which is found in the column labeled ‘Homologues Cluster ID’. The next step is to use the cluster identifier (HGDOG11214at1955251) in the ‘Orthologue Cluster ID → Genes’ template query to retrieve all pairwise gene relationships in the cluster, and save a list of the genes (Figure 2B). Finally, use the gene list in a ‘Gene ID → Protein and Coding Sequences’ template query, under the ‘Genes’ template category, to retrieve protein and coding sequences, which you can export to perform molecular evolutionary analyses (Figure 2C). Sequence lengths are provided in the query output so that you can easily select the longest protein and coding sequence of multi-transcript genes. To retrieve sequences for a non-parasitoid outgroup, you can repeat the ‘Gene ID → Homologue’ search, selecting ‘Hymenoptera’ rather than ‘Parasitoida’ as the ‘Last Common Ancestor’. In the output, note the gene id for the species you would like to use as an outgroup, and use that gene id in the ‘Gene ID → Protein and Coding Sequences’ template query. An additional example highlighting the new HGD-Ortho dataset is provided in Supplementary File 1, which shows how HymenopteraMine is used to identify *D. melanogaster* homologues and their Reactome pathways for a list of genes in *Bombus vosnesenskii*, a species that currently has little annotation information available from external resources. We also demonstrate how to programmatically use this same list of genes in the following section on the HymenopteraMine Application Programming Interface (API).

## APPLICATION PROGRAMMING INTERFACE

Although the HymenopteraMine API is not new, it has not been previously reported. HymenopteraMine leverages the web service API provided with the InterMine platform, enabling users to automate workflows and access data without using the webapp. Client library support is provided in Python, Perl, Java, JavaScript, Ruby and R (68,69). Before using the API, you should generate an API key by logging in to HymenopteraMine, going to the ‘Account Details’ page under the MyMine tab and clicking ‘Generate a new API key’. The generated token can be used in place of your user credentials in API scripts and still enables any lists generated programmatically to be saved to your MyMine account. Information to help you get started with the client API libraries is available by clicking the API tab in the HymenopteraMine navigation bar. You can also become familiar with the API by clicking ‘Perl’, ‘Python’, ‘Ruby’ or ‘Java’ in the bar near the bottom of any template query menu to retrieve automatically generated code. HymenopteraMine API examples provided in Supplementary File 1 show how to upload a list of genes and perform a template query to retrieve *D. melanogaster* homologues and their Reactome pathways.

## CITING HGD AND DATA SOURCES

You should cite this article for the use of any HGD tools, including BLAST, JBrowse/Apollo and HymenopteraMine, as well as HGD code modifications available on GitHub (https://github.com/elsiklab/). You should also cite the original genome publication and HymenopteraMine data sources for the data you used. A list of genome publications may be found by clicking ‘Genome Publications’ in the HGD navigation bar, and PubMed links are provided for all datasets on the HymenopteraMine Data Source page, accessible in the HymenopteraMine navigation bar.

## CONCLUDING REMARKS

By gathering genomic data for hymenopteran species into a single resource, HGD facilitates data reuse, meta-analysis, and cross-species comparison. We report almost triple the number of species in HGD since the previous update. To better support species that are poorly represented in external genome annotation data sources, we have generated new GO annotation and orthologue datasets for all species in HGD. For most of the species, the new HGD GO Annotation dataset makes HymenopteraMine the only publicly available web-based tool for GO enrichment analysis. The new HGD-Ortho dataset is the only web-based orthologue resource for twelve of the HGD species, and it benefits all of the included species by increasing the number of hymenoptera taxa available for comparison. We will continue to add species to HGD as genomes become available in the RefSeq division of NCBI. We encourage researchers to contact us if they have suggestions or data to contribute.

## DATA AVAILABILITY

HGD tools and data are freely available at http://HymenopteraGenome.org. Although HymenopteraMine does not require login, registering for a MyMine account allows users to save lists for future sessions and to create an API key for programmatic access. Registration is freely available and simply requires entering an email and creating a password. HymenopteraMine code is available at https://github.com/elsiklab/.

## Supplementary Material

gkab1018_Supplemental_Files

## References

[1] Elsik C.G. , Tayal A., Diesh C.M., Unni D.R., Emery M.L., Nguyen H.N., Hagen D.E. Hymenoptera Genome Database: integrating genome annotations in HymenopteraMine. Nucleic Acids Res. 2016; 44:D793–D800. 26578564 10.1093/nar/gkv1208 PMC4702858

[2] Sadd B.M. , Barribeau S.M., Bloch G., de Graaf D.C., Dearden P., Elsik C.G., Gadau J., Grimmelikhuijzen C.J., Hasselmann M., Lozier J.D. et al. The genomes of two key bumblebee species with primitive eusocial organization. Genome Biol. 2015; 16:76. 25908251 10.1186/s13059-015-0623-3 PMC4414376

[3] Rehan S.M. , Glastad K.M., Lawson S.P., Hunt B.G. The genome and methylome of a subsocial small carpenter bee, ceratina calcarata. Genome Biol. Evol. 2016; 8:1401–1410. 27048475 10.1093/gbe/evw079 PMC4898796

[4] Kapheim K.M. , Pan H., Li C., Salzberg S.L., Puiu D., Magoc T., Robertson H.M., Hudson M.E., Venkat A., Fischman B.J. et al. Social evolution. Genomic signatures of evolutionary transitions from solitary to group living. Science. 2015; 348:1139–1143. 25977371 10.1126/science.aaa4788 PMC5471836

[5] Kocher S.D. , Li C., Yang W., Tan H., Yi S.V., Yang X., Hoekstra H.E., Zhang G., Pierce N.E., Yu D.W. The draft genome of a socially polymorphic halictid bee, Lasioglossum albipes. Genome Biol. 2013; 14:R142. 24359881 10.1186/gb-2013-14-12-r142 PMC4062844

[6] Bonasio R. , Zhang G., Ye C., Mutti N.S., Fang X., Qin N., Donahue G., Yang P., Li Q., Li C. et al. Genomic comparison of the ants *Camponotus floridanus* and *Harpegnathos saltator*. Science. 2010; 329:1068–1071. 20798317 10.1126/science.1192428 PMC3772619

[7] Oxley P.R. , Ji L., Fetter-Pruneda I., McKenzie S.K., Li C., Hu H., Zhang G., Kronauer D.J. The genome of the clonal raider ant *Cerapachys biroi*. Current biology : CB. 2014; 24:451–458. 24508170 10.1016/j.cub.2014.01.018 PMC3961065

[8] Kapheim K.M. , Jones B.M., Pan H., Li C., Harpur B.A., Kent C.F., Zayed A., Ioannidis P., Waterhouse R.M., Kingwell C. et al. Developmental plasticity shapes social traits and selection in a facultatively eusocial bee. PNAS. 2020; 117:13615–13625. 32471944 10.1073/pnas.2000344117 PMC7306772

[9] Nygaard S. , Zhang G., Schiott M., Li C., Wurm Y., Hu H., Zhou J., Ji L., Qiu F., Rasmussen M. et al. The genome of the leaf-cutting ant *Acromyrmex echinatior* suggests key adaptations to advanced social life and fungus farming. Genome Res. 2011; 21:1339–1348. 21719571 10.1101/gr.121392.111 PMC3149500

[10] Suen G. , Teiling C., Li L., Holt C., Abouheif E., Bornberg-Bauer E., Bouffard P., Caldera E.J., Cash E., Cavanaugh A. et al. The genome sequence of the leaf-cutter ant Atta cephalotes reveals insights into its obligate symbiotic lifestyle. PLoS Genet. 2011; 7:e1002007. 21347285 10.1371/journal.pgen.1002007 PMC3037820

[11] Nygaard S. , Hu H., Li C., Schiott M., Chen Z., Yang Z., Xie Q., Ma C., Deng Y., Dikow R.B. et al. Reciprocal genomic evolution in the ant-fungus agricultural symbiosis. Nat. Commun. 2016; 7:12233. 27436133 10.1038/ncomms12233 PMC4961791

[12] Rubin B.E. , Moreau C.S. Comparative genomics reveals convergent rates of evolution in ant-plant mutualisms. Nat. Commun. 2016; 7:12679. 27557866 10.1038/ncomms12679 PMC5007375

[13] Xiao J.H. , Yue Z., Jia L.Y., Yang X.H., Niu L.H., Wang Z., Zhang P., Sun B.F., He S.M., Li Z. et al. Obligate mutualism within a host drives the extreme specialization of a fig wasp genome. Genome Biol. 2013; 14:R141. 24359812 10.1186/gb-2013-14-12-r141 PMC4053974

[14] Smith C.R. , Helms Cahan S., Kemena C., Brady S.G., Yang W., Bornberg-Bauer E., Eriksson T., Gadau J., Helmkampf M., Gotzek D. et al. How do genomes create novel phenotypes? Insights from the loss of the worker caste in ant social parasites. Mol. Biol. Evol. 2015; 32:2919–2931. 26226984 10.1093/molbev/msv165 PMC4651238

[15] Geib S.M. , Liang G.H., Murphy T.D., Sim S.B. Whole genome sequencing of the braconid parasitoid wasp fopius arisanus, an important biocontrol agent of pest tepritid fruit flies. G3 (Bethesda). 2017; 7:2407–2411. 28584080 10.1534/g3.117.040741 PMC5555450

[16] Lindsey A.R.I. , Kelkar Y.D., Wu X., Sun D., Martinson E.O., Yan Z., Rugman-Jones P.F., Hughes D.S.T., Murali S.C., Qu J. et al. Comparative genomics of the miniature wasp and pest control agent *Trichogramma pretiosum*. BMC Biol. 2018; 16:54. 29776407 10.1186/s12915-018-0520-9 PMC5960102

[17] Werren J.H. , Richards S., Desjardins C.A., Niehuis O., Gadau J., Colbourne J.K., Nasonia Genome Working G., Werren J.H., Richards S., Desjardins C.A. et al. Functional and evolutionary insights from the genomes of three parasitoid *Nasonia* species. Science. 2010; 327:343–348. 20075255 10.1126/science.1178028 PMC2849982

[18] Dhaygude K. , Nair A., Johansson H., Wurm Y., Sundstrom L. The first draft genomes of the ant *Formica exsecta*, and its *Wolbachia endosymbiont* reveal extensive gene transfer from endosymbiont to host. BMC Genomics. 2019; 20:301. 30991952 10.1186/s12864-019-5665-6 PMC6469114

[19] Burke G.R. , Walden K.K.O., Whitfield J.B., Robertson H.M., Strand M.R. Whole genome sequence of the parasitoid wasp microplitis demolitor that harbors an endogenous virus mutualist. G3 (Bethesda). 2018; 8:2875–2880. 30018085 10.1534/g3.118.200308 PMC6118312

[20] Schrader L. , Kim J.W., Ence D., Zimin A., Klein A., Wyschetzki K., Weichselgartner T., Kemena C., Stokl J., Schultner E. et al. Transposable element islands facilitate adaptation to novel environments in an invasive species. Nat. Commun. 2014; 5:5495. 25510865 10.1038/ncomms6495 PMC4284661

[21] Tvedte E.S. , Walden K.K.O., McElroy K.E., Werren J.H., Forbes A.A., Hood G.R., Logsdon J.M., Feder J.L., Robertson H.M. Genome of the parasitoid wasp diachasma alloeum, an emerging model for ecological speciation and transitions to asexual reproduction. Genome Biol Evol. 2019; 11:2767–2773. 31553440 10.1093/gbe/evz205 PMC6781843

[22] Patalano S. , Vlasova A., Wyatt C., Ewels P., Camara F., Ferreira P.G., Asher C.L., Jurkowski T.P., Segonds-Pichon A., Bachman M. et al. Molecular signatures of plastic phenotypes in two eusocial insect species with simple societies. PNAS. 2015; 112:13970–13975. 26483466 10.1073/pnas.1515937112 PMC4653166

[23] Dalla Benetta E. , Antoshechkin I., Yang T., Nguyen H.Q.M., Ferree P.M., Akbari O.S. Genome elimination mediated by gene expression from a selfish chromosome. Sci. Adv. 2020; 6:eaaz9808. 32284986 10.1126/sciadv.aaz9808 PMC7124933

[24] Beadle K. , Singh K.S., Troczka B.J., Randall E., Zaworra M., Zimmer C.T., Hayward A., Reid R., Kor L., Kohler M. et al. Genomic insights into neonicotinoid sensitivity in the solitary bee Osmia bicornis. PLos Genet. 2019; 15:e1007903. 30716069 10.1371/journal.pgen.1007903 PMC6375640

[25] Inouye D.W. Levin S.A. Pollinators, Role of. Encyclopedia of Biodiversity. 2013; 2nd edn Waltham Academic Press 140–146.

[26] Buels R. , Yao E., Diesh C.M., Hayes R.D., Munoz-Torres M., Helt G., Goodstein D.M., Elsik C.G., Lewis S.E., Stein L. et al. JBrowse: a dynamic web platform for genome visualization and analysis. Genome Biol. 2016; 17:66. 27072794 10.1186/s13059-016-0924-1 PMC4830012

[27] Dunn N.A. , Unni D.R., Diesh C., Munoz-Torres M., Harris N.L., Yao E., Rasche H., Holmes I.H., Elsik C.G., Lewis S.E. Apollo: democratizing genome annotation. PLoS Comput. Biol. 2019; 15:e1006790. 30726205 10.1371/journal.pcbi.1006790 PMC6380598

[28] Altschul S.F. , Gish W., Miller W., Myers E.W., Lipman D.J. Basic local alignment search tool. J. Mol. Biol. 1990; 215:403–410. 2231712 10.1016/S0022-2836(05)80360-2

[29] Priyam A. , Woodcroft B.J., Rai V., Moghul I., Munagala A., Ter F., Chowdhary H., Pieniak I., Maynard L.J., Gibbins M.A. et al. Sequenceserver: a modern graphical user interface for custom BLAST databases. Mol. Biol. Evol. 2019; 36:2922–2924. 31411700 10.1093/molbev/msz185 PMC6878946

[30] Smith R.N. , Aleksic J., Butano D., Carr A., Contrino S., Hu F., Lyne M., Lyne R., Kalderimis A., Rutherford K. et al. InterMine: a flexible data warehouse system for the integration and analysis of heterogeneous biological data. Bioinformatics. 2012; 28:3163–3165. 23023984 10.1093/bioinformatics/bts577 PMC3516146

[31] O’Leary N.A. , Wright M.W., Brister J.R., Ciufo S., Haddad D., McVeigh R., Rajput B., Robbertse B., Smith-White B., Ako-Adjei D. et al. Reference sequence (RefSeq) database at NCBI: current status, taxonomic expansion, and functional annotation. Nucleic Acids Res. 2016; 44:D733–D745. 26553804 10.1093/nar/gkv1189 PMC4702849

[32] UniProt Consortium. UniProt: the universal protein knowledgebase in 2021. Nucleic Acids Res. 2021; 49:D480–D489. 33237286 10.1093/nar/gkaa1100 PMC7778908

[33] Blum M. , Chang H.Y., Chuguransky S., Grego T., Kandasaamy S., Mitchell A., Nuka G., Paysan-Lafosse T., Qureshi M., Raj S. et al. The InterPro protein families and domains database: 20 years on. Nucleic Acids Res. 2021; 49:D344–D354. 33156333 10.1093/nar/gkaa977 PMC7778928

[34] Zdobnov E.M. , Kuznetsov D., Tegenfeldt F., Manni M., Berkeley M., Kriventseva E.V. OrthoDB in 2020: evolutionary and functional annotations of orthologs. Nucleic Acids Res. 2021; 49:D389–D393. 33196836 10.1093/nar/gkaa1009 PMC7779051

[35] Kanehisa M. , Furumichi M., Sato Y., Ishiguro-Watanabe M., Tanabe M. KEGG: integrating viruses and cellular organisms. Nucleic Acids Res. 2021; 49:D545–D551. 33125081 10.1093/nar/gkaa970 PMC7779016

[36] Sayers E.W. , Beck J., Bolton E.E., Bourexis D., Brister J.R., Canese K., Comeau D.C., Funk K., Kim S., Klimke W. et al. Database resources of the national center for biotechnology information. Nucleic Acids Res. 2021; 49:D10–D17. 33095870 10.1093/nar/gkaa892 PMC7778943

[37] Oughtred R. , Rust J., Chang C., Breitkreutz B.J., Stark C., Willems A., Boucher L., Leung G., Kolas N., Zhang F. et al. The BioGRID database: a comprehensive biomedical resource of curated protein, genetic, and chemical interactions. Protein Sci. 2021; 30:187–200. 33070389 10.1002/pro.3978 PMC7737760

[38] Jassal B. , Matthews L., Viteri G., Gong C., Lorente P., Fabregat A., Sidiropoulos K., Cook J., Gillespie M., Haw R. et al. The reactome pathway knowledgebase. Nucleic Acids Res. 2020; 48:D498–D503. 31691815 10.1093/nar/gkz1031 PMC7145712

[39] Orchard S. , Ammari M., Aranda B., Breuza L., Briganti L., Broackes-Carter F., Campbell N.H., Chavali G., Chen C., del-Toro N. et al. The MIntAct project–IntAct as a common curation platform for 11 molecular interaction databases. Nucleic Acids Res. 2014; 42:D358–D363. 24234451 10.1093/nar/gkt1115 PMC3965093

[40] Elsik C.G. , Tayal A., Unni D.R., Burns G.W., Hagen D.E. Hymenoptera genome database: using hymenopteramine to enhance genomic studies of hymenopteran insects. Methods Mol. Biol. 2018; 1757:513–556. 29761469 10.1007/978-1-4939-7737-6_17

[41] Smith C.D. , Zimin A., Holt C., Abouheif E., Benton R., Cash E., Croset V., Currie C.R., Elhaik E., Elsik C.G. et al. Draft genome of the globally widespread and invasive Argentine ant (*Linepithema humile*). PNAS. 2011; 108:5673–5678. 21282631 10.1073/pnas.1008617108 PMC3078359

[42] Gao Q. , Xiong Z., Larsen R.S., Zhou L., Zhao J., Ding G., Zhao R., Liu C., Ran H., Zhang G. High-quality chromosome-level genome assembly and full-length transcriptome analysis of the pharaoh ant *Monomorium pharaonis*. Gigascience. 2020; 9:giaa143. 33319913 10.1093/gigascience/giaa143 PMC7736795

[43] Smith C.R. , Smith C.D., Robertson H.M., Helmkampf M., Zimin A., Yandell M., Holt C., Hu H., Abouheif E., Benton R. et al. Draft genome of the red harvester ant Pogonomyrmex barbatus. PNAS. 2011; 108:5667–5672. 21282651 10.1073/pnas.1007901108 PMC3078412

[44] Wurm Y. , Wang J., Riba-Grognuz O., Corona M., Nygaard S., Hunt B.G., Ingram K.K., Falquet L., Nipitwattanaphon M., Gotzek D. et al. The genome of the fire ant Solenopsis invicta. PNAS. 2011; 108:5679–5684. 21282665 10.1073/pnas.1009690108 PMC3078418

[45] Wang Z.L. , Zhu Y.Q., Yan Q., Yan W.Y., Zheng H.J., Zeng Z.J. A chromosome-scale assembly of the asian honeybee apis cerana Genome. Front Genet. 2020; 11:279. 32292419 10.3389/fgene.2020.00279 PMC7119468

[46] Fouks B. , Brand P., Nguyen H.N., Herman J., Camara F., Ence D., Hagen D.E., Hoff K.J., Nachweide S., Romoth L. et al. The genomic basis of evolutionary differentiation among honey bees. Genome Res. 2021; 31:1203–1215. 10.1101/gr.272310.120 PMC8256857 33947700

[47] Wallberg A. , Bunikis I., Pettersson O.V., Mosbech M.B., Childers A.K., Evans J.D., Mikheyev A.S., Robertson H.M., Robinson G.E., Webster M.T. A hybrid de novo genome assembly of the honeybee, *Apis mellifera*, with chromosome-length scaffolds. BMC Genomics. 2019; 20:275. 30961563 10.1186/s12864-019-5642-0 PMC6454739

[48] Heraghty S.D. , Sutton J.M., Pimsler M.L., Fierst J.L., Strange J.P., Lozier J.D. De novo genome assemblies for three north american bumble bee species: *Bombus bifarius*, *Bombus vancouverensis*, and *Bombus vosnesenskii*. G3 (Bethesda). 2020; 10:2585–2592. 32586847 10.1534/g3.120.401437 PMC7407468

[49] Kapheim K.M. , Pan H., Li C., Blatti C. 3rd, Harpur B.A., Ioannidis P., Jones B.M., Kent C.F., Ruzzante L., Sloofman L. et al. Draft genome assembly and population genetics of an agricultural pollinator, the solitary alkali bee (Halictidae: Nomia melanderi). G3 (Bethesda). 2019; 9:625–634. 30642875 10.1534/g3.118.200865 PMC6404593

[50] Robertson H.M. , Waterhouse R.M., Walden K.K.O., Ruzzante L., Reijnders M., Coates B.S., Legeai F., Gress J.C., Biyiklioglu S., Weaver D.K. et al. Genome sequence of the wheat stem sawfly, cephus cinctus, representing an early-branching lineage of the hymenoptera, illuminates evolution of hymenopteran chemoreceptors. Genome Biol Evol. 2018; 10:2997–3011. 30335145 10.1093/gbe/evy232 PMC6250288

[51] Linnen C.R. , O’Quin C.T., Shackleford T., Sears C.R., Lindstedt C. Genetic basis of body color and spotting pattern in redheaded pine sawfly larvae (neodiprion lecontei). Genetics. 2018; 209:291–305. 29496749 10.1534/genetics.118.300793 PMC5937194

[52] Oeyen J.P. , Baa-Puyoulet P., Benoit J.B., Beukeboom L.W., Bornberg-Bauer E., Buttstedt A., Calevro F., Cash E.I., Chao H., Charles H. et al. Sawfly genomes reveal evolutionary acquisitions that fostered the mega-radiation of parasitoid and eusocial hymenoptera. Genome Biol Evol. 2020; 12:1099–1188. 32442304 10.1093/gbe/evaa106 PMC7455281

[53] Standage D.S. , Berens A.J., Glastad K.M., Severin A.J., Brendel V.P., Toth A.L. Genome, transcriptome and methylome sequencing of a primitively eusocial wasp reveal a greatly reduced DNA methylation system in a social insect. Mol. Ecol. 2016; 25:1769–1784. 26859767 10.1111/mec.13578

[54] Thomas G.W.C. , Dohmen E., Hughes D.S.T., Murali S.C., Poelchau M., Glastad K., Anstead C.A., Ayoub N.A., Batterham P., Bellair M. et al. Gene content evolution in the arthropods. Genome Biol. 2020; 21:15. 31969194 10.1186/s13059-019-1925-7 PMC6977273

[55] Solares E.A. , Chakraborty M., Miller D.E., Kalsow S., Hall K., Perera A.G., Emerson J.J., Hawley R.S. Rapid low-cost assembly of the drosophila melanogaster reference genome using low-coverage, long-read sequencing. G3 (Bethesda). 2018; 8:3143–3154. 30018084 10.1534/g3.118.200162 PMC6169397

[56] Huntley R.P. , Sawford T., Mutowo-Meullenet P., Shypitsyna A., Bonilla C., Martin M.J., O’Donovan C. The GOA database: gene ontology annotation updates for 2015. Nucleic Acids Res. 2015; 43:D1057–D1063. 25378336 10.1093/nar/gku1113 PMC4383930

[57] Jones P. , Binns D., Chang H.Y., Fraser M., Li W., McAnulla C., McWilliam H., Maslen J., Mitchell A., Nuka G. et al. InterProScan 5: genome-scale protein function classification. Bioinformatics. 2014; 30:1236–1240. 24451626 10.1093/bioinformatics/btu031 PMC3998142

[58] Letunic I. , Khedkar S., Bork P. SMART: recent updates, new developments and status in 2020. Nucleic Acids Res. 2021; 49:D458–D460. 33104802 10.1093/nar/gkaa937 PMC7778883

[59] Madera M. , Vogel C., Kummerfeld S.K., Chothia C., Gough J. The SUPERFAMILY database in 2004: additions and improvements. Nucleic Acids Res. 2004; 32:D235–D239. 14681402 10.1093/nar/gkh117 PMC308851

[60] Mi H. , Ebert D., Muruganujan A., Mills C., Albou L.P., Mushayamaha T., Thomas P.D. PANTHER version 16: a revised family classification, tree-based classification tool, enhancer regions and extensive API. Nucleic Acids Res. 2021; 49:D394–D403. 33290554 10.1093/nar/gkaa1106 PMC7778891

[61] Mistry J. , Chuguransky S., Williams L., Qureshi M., Salazar G.A., Sonnhammer E.L.L., Tosatto S.C.E., Paladin L., Raj S., Richardson L.J. et al. Pfam: the protein families database in 2021. Nucleic Acids Res. 2021; 49:D412–D419. 33125078 10.1093/nar/gkaa913 PMC7779014

[62] Pearson W.R. Finding protein and nucleotide similarities with FASTA. Curr Protoc Bioinformatics. 2016; 53:3.9.1–3.9.25. 27010337 10.1002/0471250953.bi0309s53 PMC5072362

[63] Larkin A. , Marygold S.J., Antonazzo G., Attrill H., Dos Santos G., Garapati P.V., Goodman J.L., Gramates L.S., Millburn G., Strelets V.B. et al. FlyBase: updates to the *Drosophila melanogaster* knowledge base. Nucleic Acids Res. 2021; 49:D899–D907. 33219682 10.1093/nar/gkaa1026 PMC7779046

[64] Huntley R.P. , Sawford T., Martin M.J., O’Donovan C. Understanding how and why the Gene Ontology and its annotations evolve: the GO within UniProt. Gigascience. 2014; 3:4. 24641996 10.1186/2047-217X-3-4 PMC3995153

[65] Ashburner M. , Ball C.A., Blake J.A., Botstein D., Butler H., Cherry J.M., Davis A.P., Dolinski K., Dwight S.S., Eppig J.T. et al. Gene ontology: tool for the unification of biology. The gene ontology consortium. Nat. Genet. 2000; 25:25–29. 10802651 10.1038/75556 PMC3037419

[66] Gene Ontology Consortium The Gene Ontology resource: enriching a GOld mine. Nucleic Acids Res. 2021; 49:D325–D334. 33290552 10.1093/nar/gkaa1113 PMC7779012

[67] Castillo-Davis C.I. , Hartl D.L. GeneMerge–post-genomic analysis, data mining, and hypothesis testing. Bioinformatics. 2003; 19:891–892. 12724301 10.1093/bioinformatics/btg114

[68] Kalderimis A. , Lyne R., Butano D., Contrino S., Lyne M., Heimbach J., Hu F., Smith R., Stepan R., Sullivan J. et al. InterMine: extensive web services for modern biology. Nucleic Acids Res. 2014; 42:W468–W472. 24753429 10.1093/nar/gku301 PMC4086141

[69] Kyritsis K.A. , Wang B., Sullivan J., Lyne R., Micklem G. InterMineR: an R package for InterMine databases. Bioinformatics. 2019; 35:3206–3207. 30668641 10.1093/bioinformatics/btz039 PMC6736411

