# Supplementary material for: Hymenoptera Genome Database: new genomes and annotation datasets for improved go enrichment and orthologue analyses
Source: Nucleic Acids Res. 2021 Nov 8;50(D1):D1032–9. doi: 10.1093/nar/gkab1018 (PMC8728238; doi:10.1093/nar/gkab1018)
Supplement: gkab1018_Supplemental_Files [file gkab1018_supplemental_files.zip › Supplementary_File_1.pdf]

Supplementary File 1: HymenopteraMine Examples

Part I: HymenopteraMine WebApp

This example demonstrates the use of the List Tool to upload a list of *Bombus vosnesenskii* gene identifiers, perform Gene Ontology (GO) enrichment analysis and use a template query to retrieve *D. melanogaster* homologues and their Reactome pathways. This example uses the gene identifiers provided in Supplementary File 2.

Click the ‘Lists’ tab in the HymenopteraMine navigation bar. This will bring you to the List Upload or List View page. If necessary, toggle to the List Upload page by clicking ‘Upload’ at the left end of the brown bar just under the main navigation bar (Figure S1).

In the List Upload menu (Figure S1), make sure ‘Gene’ is selected as the ‘Type’ and select ‘B. vosnesenskii’ as the organism. Paste the list of genes into the text box. This menu also allows you to upload a list from a txt formatted file with a single column of identifiers. Click ‘Create List’.

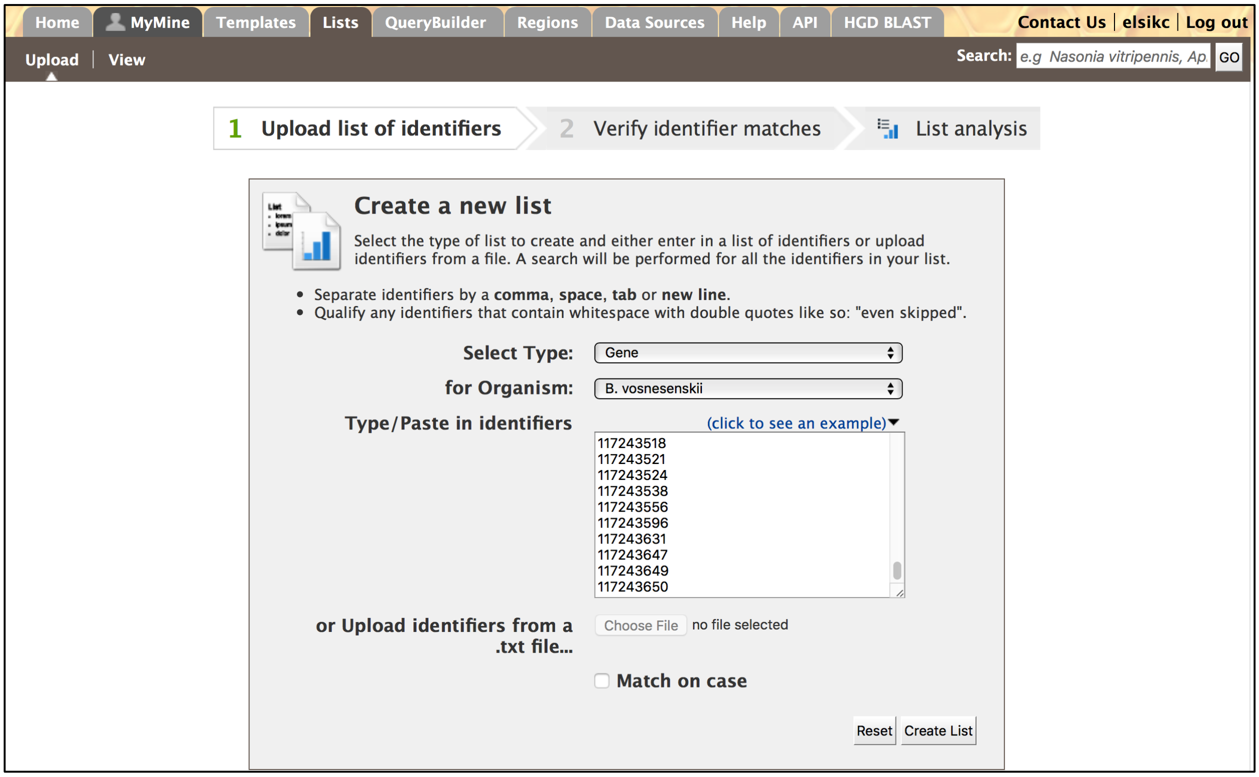

Figure S1. The List Upload page, which allows you to upload a list of identifiers.

Prior to saving the final list, a lookup is performed to verify that the identifiers are in the database. A preliminary results page (Figure S2) shows the number of identifiers uploaded and found in the database. If any uploaded identifiers are missing from the database, they are shown at the bottom of the page. In this example all identifiers are verified to be present in the database. The identifiers found are shown in a table for you to preview. Prior to clicking ‘Save a list 505 genes’, you can modify the default name for the list in the box under ‘Choose a name for the list’.

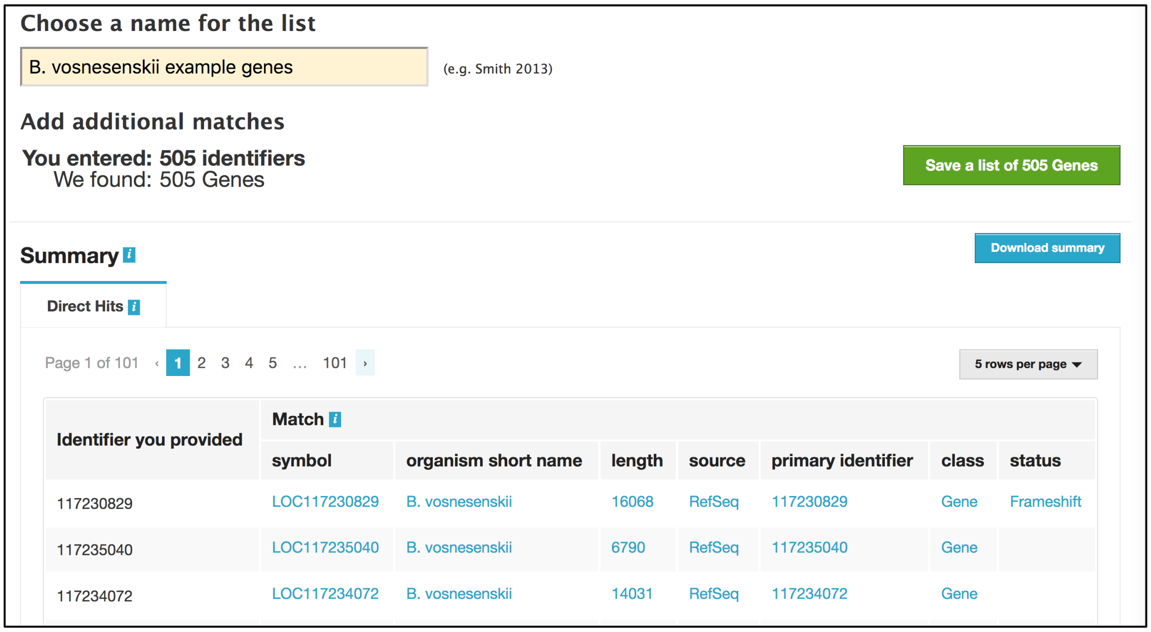

Figure S2. The identifier verification page. If any identifiers were missing, they would be listed at the bottom of this page (not shown).

After saving the list, the List Analysis page appears (Figure S3). The genes are listed in a table with a format similar to any HymenopteraMine query output, with summary information, including the gene source, chromosome location, gene symbol and gene name (if available). The column ‘Gene Status’ has entries for ‘ambiguous’ (genes that have both protein-coding and non-coding transcripts) and genes in which protein-coding sequence have frameshifts due to genome assembly issues. In most cases there is ‘No Value’ in this column.

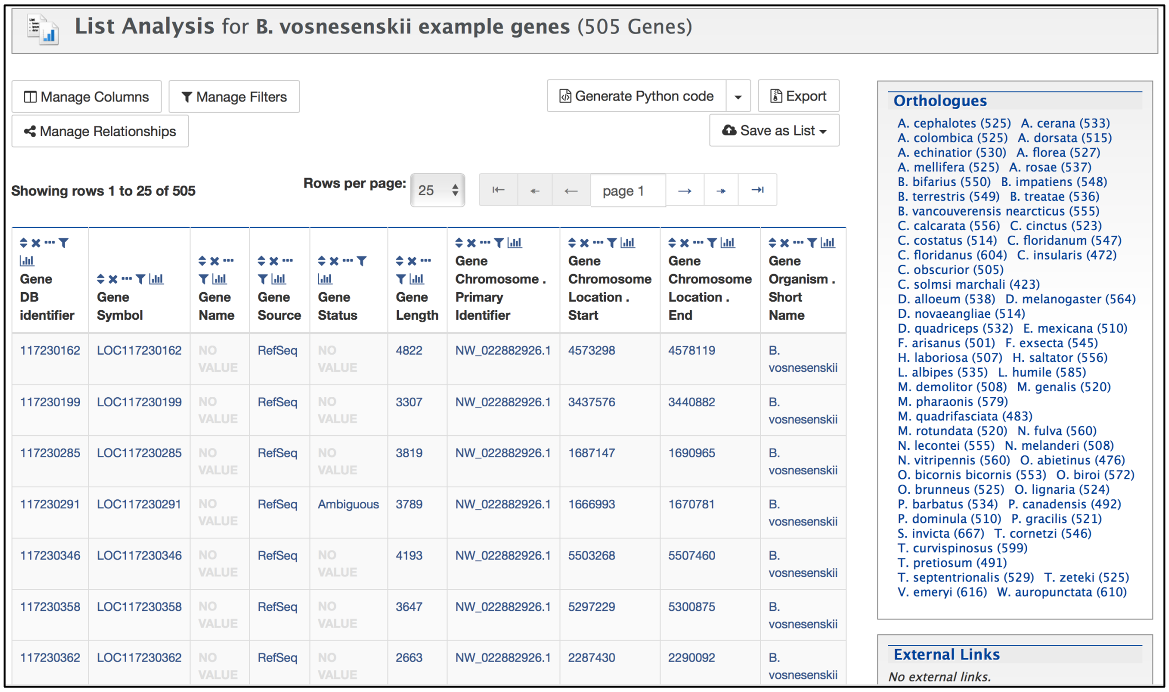

Figure S3. The List Analysis page (top part), showing the uploaded identifiers and associated information. The information provided depends on the data type of the identifiers uploaded.

After scrolling down the List Analysis page, you will see gene set enrichment widgets. All species in HymenopteraMine have GO annotations, and GO enrichment is automatically performed when saving a gene list (Figure S4). The enrichment widget has pulldown menus that allow you to modify the test correction, maximum p-value, and ontology (i.e. Biological Process, Molecular Function and Cellular Component). By default, the background population is all GO terms in the database for that species. You can use the List Tool to upload a list of genes representing a more specific background population, for example, a list of all genes expressed in an expression experiment. Once your custom background gene list is saved, it will be available in a pulldown menu that appears when you click ‘Change’ under ‘Background population’. Two species in HymenopteraMine (*Apis mellifera* and *Nasonia vitripennis*) have more than one gene set with GO annotation in HymenopteraMine, so for these species it is imperative that you change the background population for the specific gene set you are investigating. However, *B. vosnesenskii* has only one gene set, so for this example it is not necessary to change the background population. Clicking ‘View’ in the widget opens a new tab with a table output of all genes that have enriched terms, along with the terms. The ‘Download’ button allows you to download a tab-delimited file with the enrichment results, including the gene identifiers, GO terms and p-values.

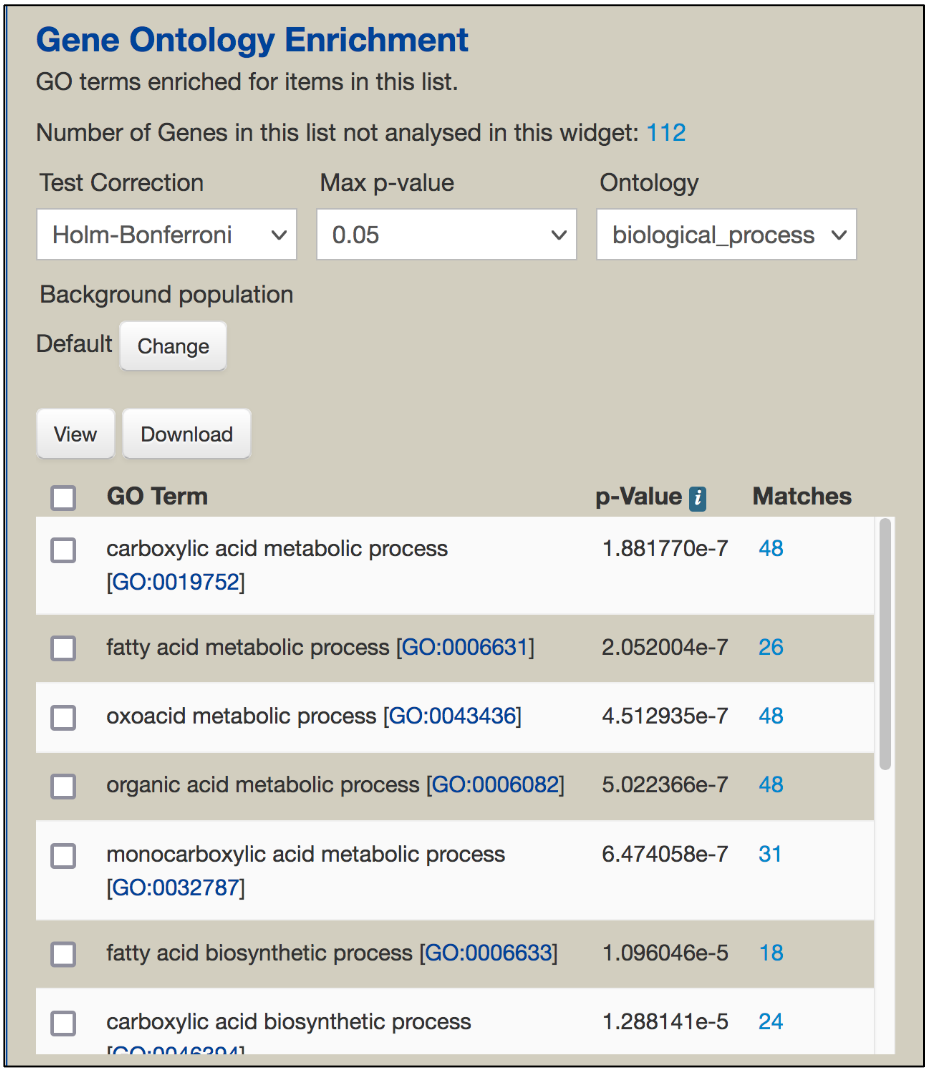

Figure S4. GO enrichment widget. HymenopteraMine has similar enrichment widgets for pathways and publications.

Once you have finished viewing the GO enrichment, you can go back to the top of the page and toggle to the List View page by clicking ‘View’ in the brown bar under the navigation bar. The List View page (Figure S5) shows your saved lists, and provides tools to perform set operations, such as intersection, to produce new lists. The lists you have saved are shown with a mauve background. Lists available to all users have a white background. These are lists of the *A. mellifera* and *N. vitripennis* gene sets, provided so that they can be used as the background population when performing gene set enrichment for those species, as described above. If you click a list name, you will go back to the List Analysis page, and enrichment will be re-computed.

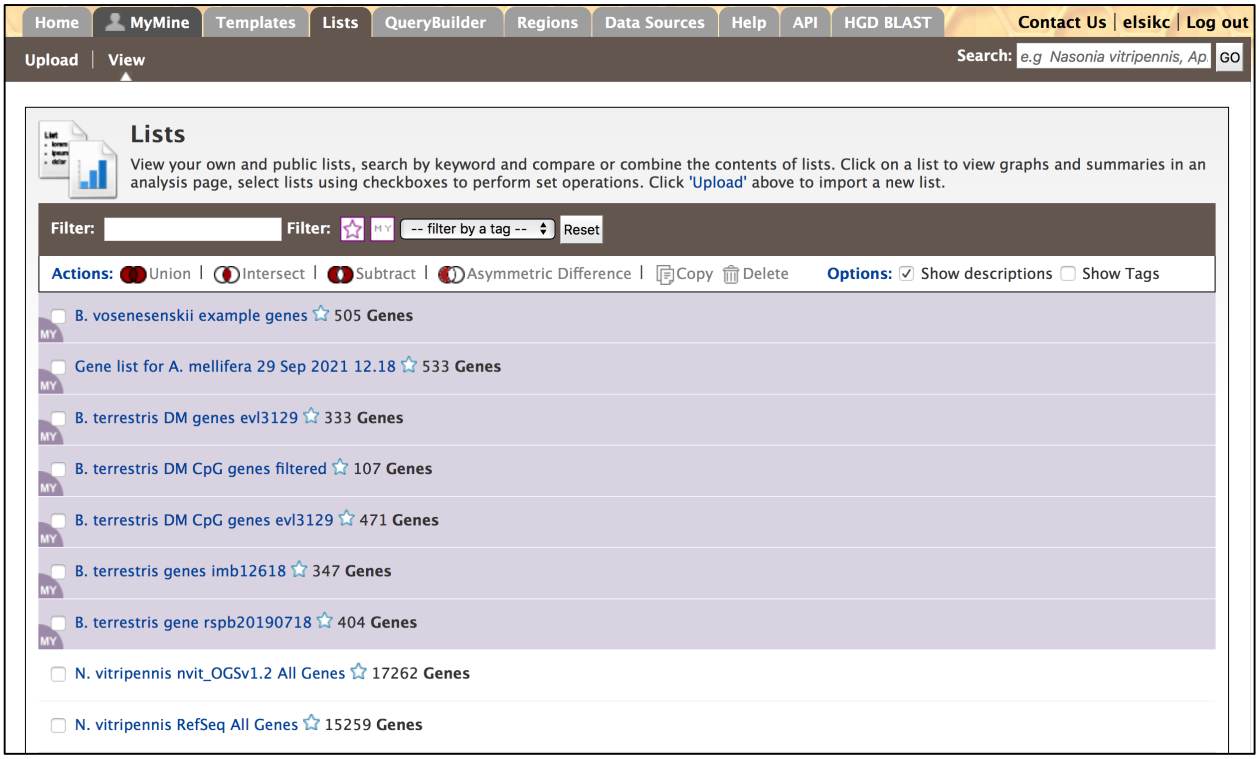

Figure S5. List View page.

The next step in this example is to run a template query using your saved list as input. Click on ‘Home’ in the navigation bar to go back to the HymenopteraMine home page. About halfway down the page is a bar with tabs for template query categories (Figure S6). Click on the ‘Homology’ tab to find the template query ‘Gene ID → D. melanogaster homologues and their Reactome pathways’. This query can also be found under the ‘Function’ category. The order of template queries shown changes according to frequency of use. If you do not see the query listed, click ‘More queries’ to see the full list for that category. Click the name of the query ‘Gene ID → D. melanogaster homologues and their Reactome pathways’ to open the query menu.

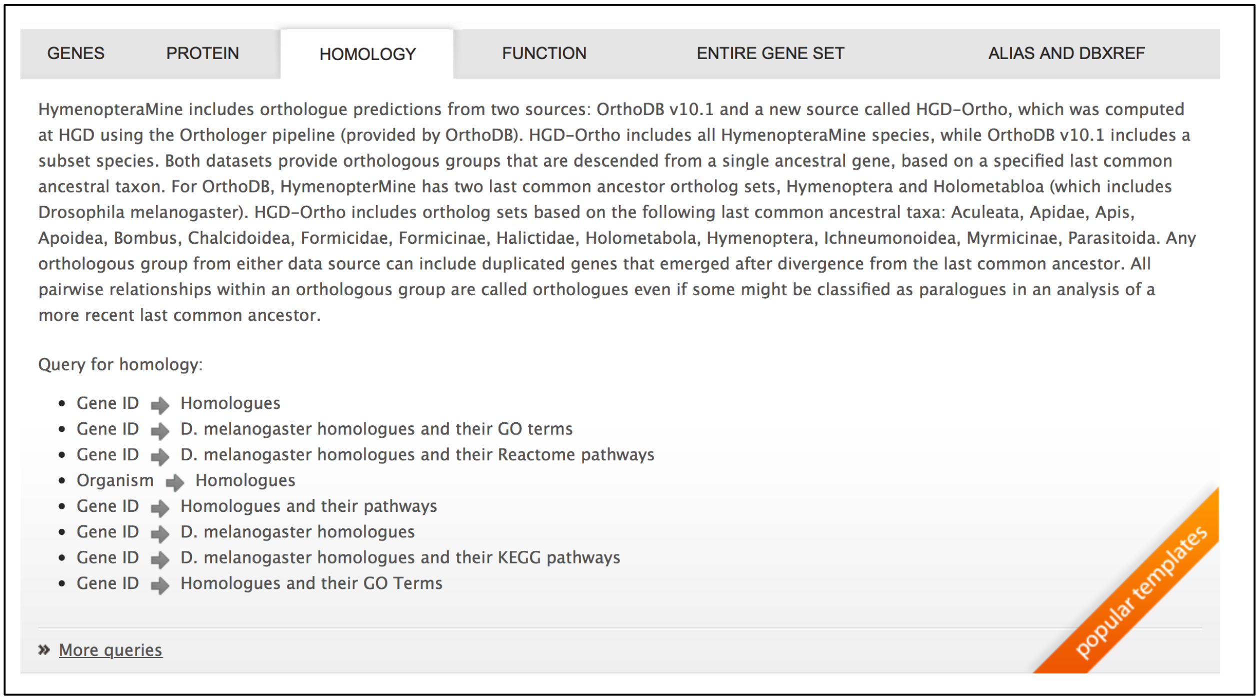

Figure S6. Template category bar, showing the ‘Homology’ category.

In the menu for ‘Gene ID → D. melanogaster homologues and their Reactome pathways’ (Figure S7), you can ignore the pre-entered example gene identifier. Instead, check the box next to “constrain to be” and leave it set to ‘IN’. Select your saved list in the pulldown menu that becomes available, and then click ‘Show Results’.

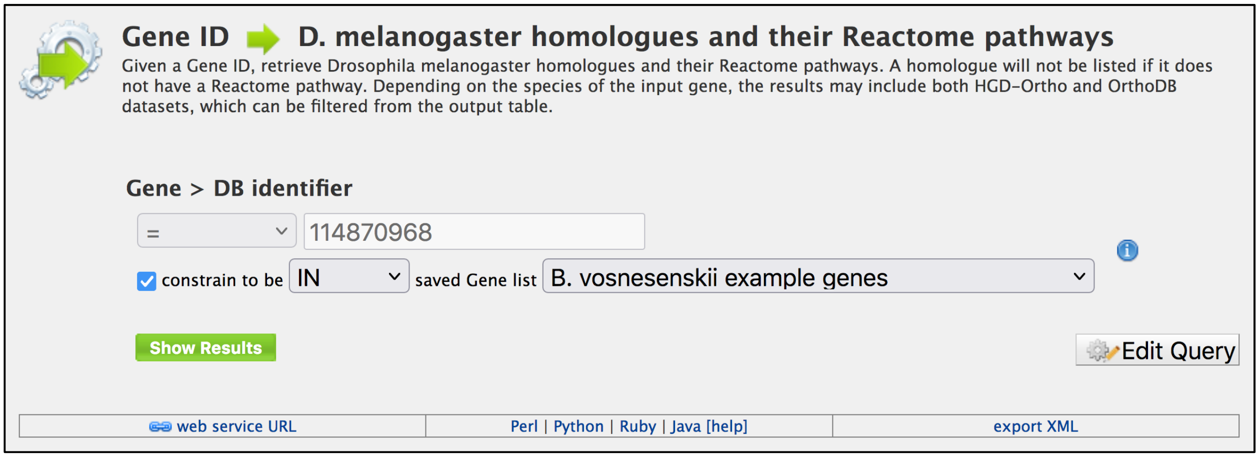

Figure S7. Menu for the ‘Gene ID → D. melanogaster and their Reactome pathways’ query.

This query may take a few moments, because it requires joining multiple datasets. The output (Figure S8) is provided as a table that can be modified using icons above each column. The small arrows allow sorting by a column in ascending or descending order. Clicking the ‘x’ deletes a column. Clicking ‘…’ hides a column from view. The funnel icon allows filtering based on numeric values. The histogram icon allows filtering based on categorical values. The ‘Manage Columns’ button above the table, to the left, allows you to reorder the columns and to add new columns. The ‘Manage Filters’ button provides the ability to add new filters.

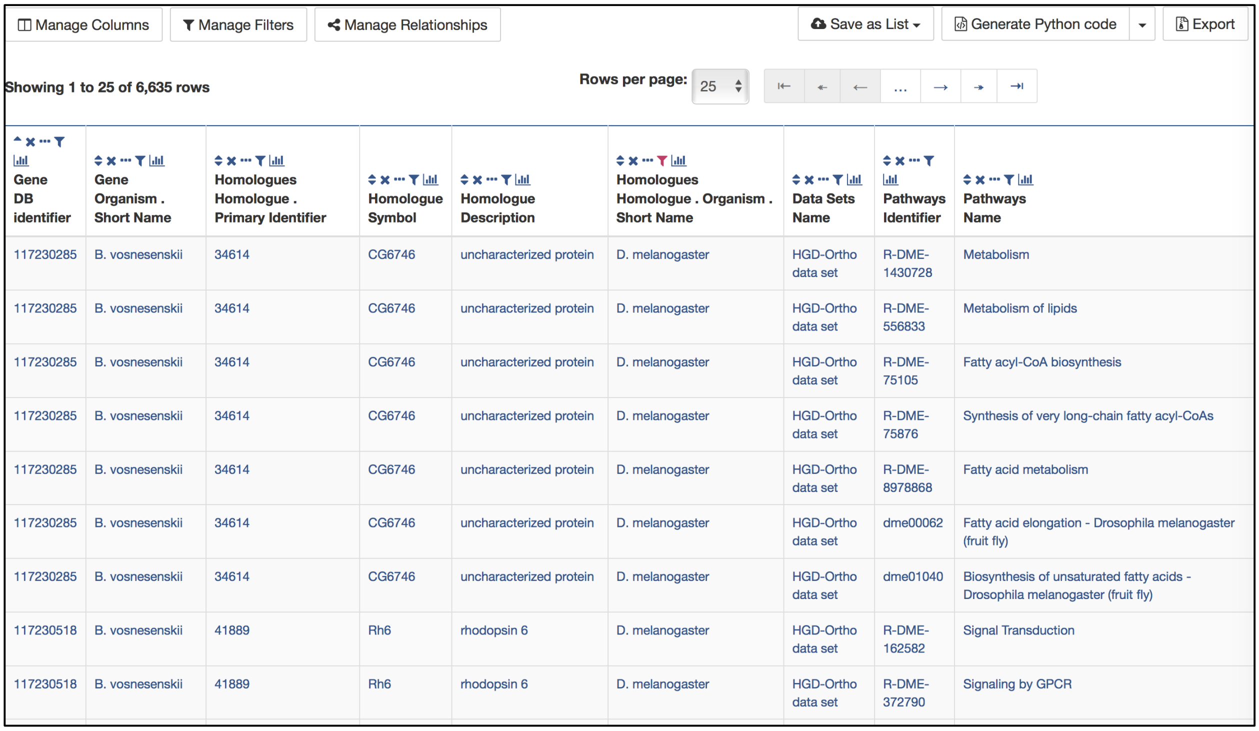

Gene DB identifier	Gene Organism . Short Name	Homologues Homologue . Primary Identifier	Homologue Symbol	Homologue Description	Homologues Homologue . Organism . Short Name	Data Sets Name	Pathways Identifier	Pathways Name
117230285	B. vosnesenskii	34614	CG6746	uncharacterized protein	D. melanogaster	HGD-Ortho data set	R-DME-1430728	Metabolism
117230285	B. vosnesenskii	34614	CG6746	uncharacterized protein	D. melanogaster	HGD-Ortho data set	R-DME-556833	Metabolism of lipids
117230285	B. vosnesenskii	34614	CG6746	uncharacterized protein	D. melanogaster	HGD-Ortho data set	R-DME-75105	Fatty acyl-CoA biosynthesis
117230285	B. vosnesenskii	34614	CG6746	uncharacterized protein	D. melanogaster	HGD-Ortho data set	R-DME-75876	Synthesis of very long-chain fatty acyl-CoAs
117230285	B. vosnesenskii	34614	CG6746	uncharacterized protein	D. melanogaster	HGD-Ortho data set	R-DME-8978868	Fatty acid metabolism
117230285	B. vosnesenskii	34614	CG6746	uncharacterized protein	D. melanogaster	HGD-Ortho data set	dme00062	Fatty acid elongation - Drosophila melanogaster (fruit fly)
117230285	B. vosnesenskii	34614	CG6746	uncharacterized protein	D. melanogaster	HGD-Ortho data set	dme01040	Biosynthesis of unsaturated fatty acids - Drosophila melanogaster (fruit fly)
117230518	B. vosnesenskii	41889	Rh6	rhodopsin 6	D. melanogaster	HGD-Ortho data set	R-DME-162582	Signal Transduction
117230518	B. vosnesenskii	41889	Rh6	rhodopsin 6	D. melanogaster	HGD-Ortho data set	R-DME-372790	Signaling by GPCR

Figure S8. Template query output. The table can be modified using icons at the top of each column, as well as ‘Manage Columns’, ‘Manage Filters’ and ‘Manage Relationships’.

If you would like to retrieve homologues even if they do not have pathways, use the ‘Manage Relationships’ menu to make pathways optional (Figure S9). Click ‘Optional’ next to ‘Gene >> Homologues >> Homologue >> Pathways’. When you make a relationship optional, the output format changes so that the optional data type is shown as embedded tables (Figure S10), which can be expanded (Figures S11). An ‘Undo’ button lets you undo any changes made to a table. The ‘Export’ button allows you to export the entire table or selected columns from the table (Figure S12).

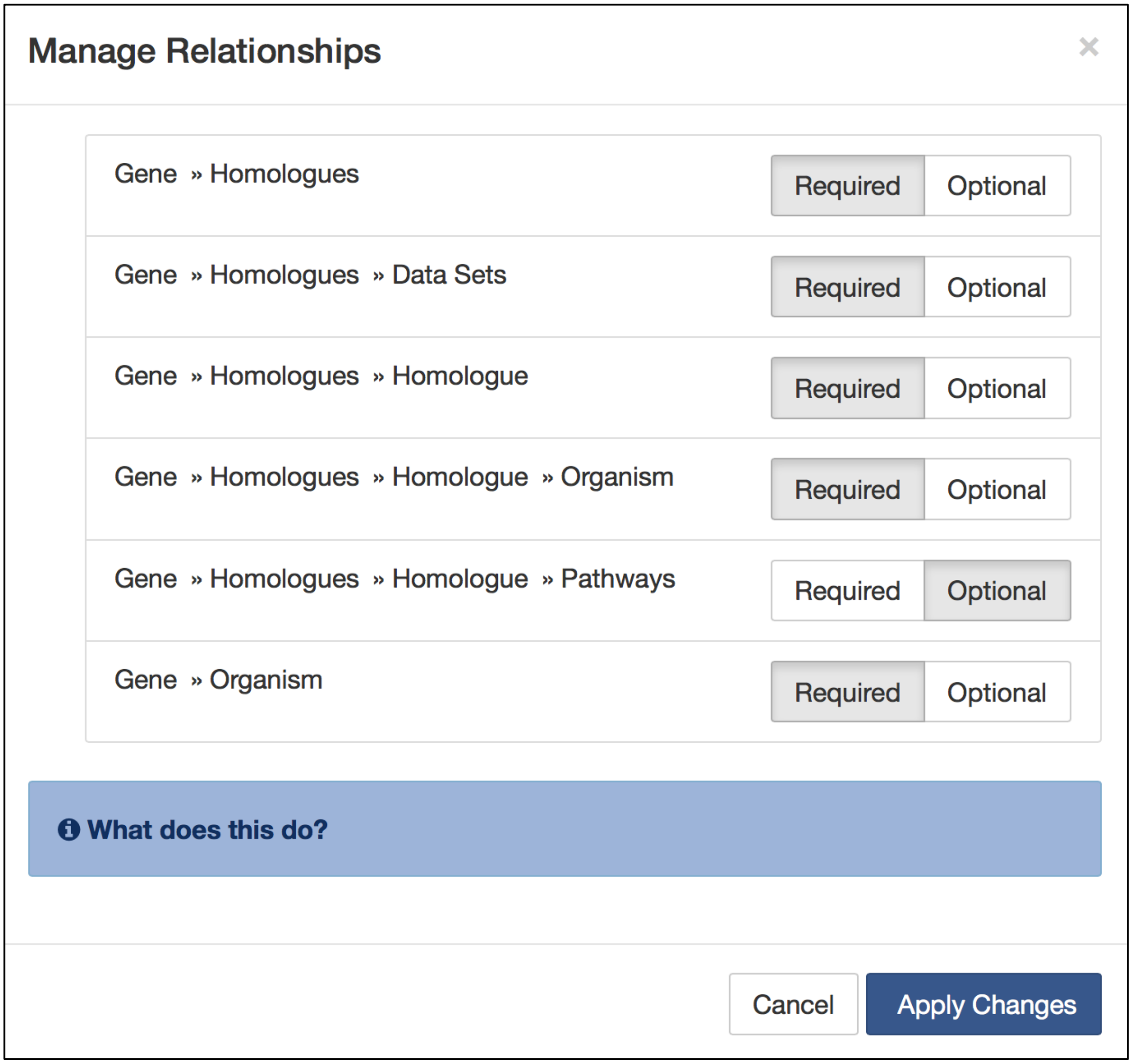

Figure S9. The ‘Manage Relationships’ menu. By clicking ‘Optional’ next to ‘Gene >> Homologues >> Homologue >> Pathways’, homologues will appear in the table even if they do not have any pathways.

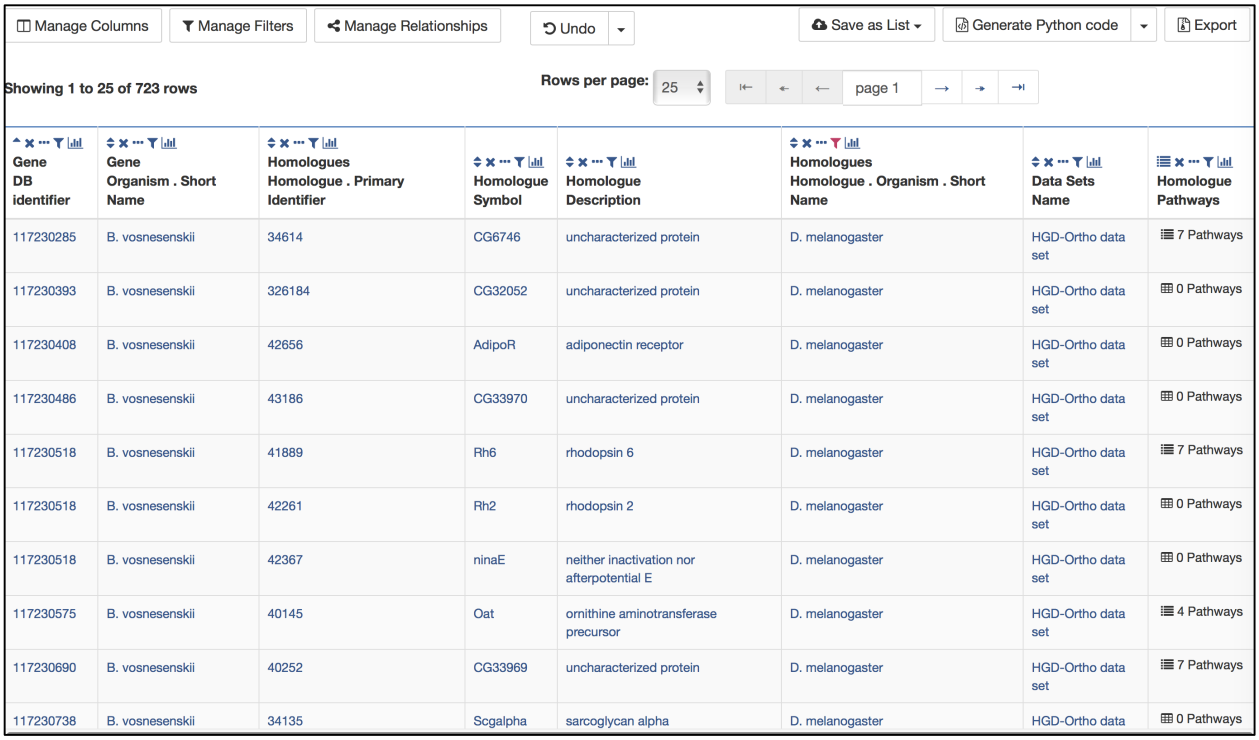

Manage Columns | Manage Filters | Manage Relationships | Undo | Save as List | Generate Python code | Export

Showing 1 to 25 of 723 rows

Rows per page: 25 | page 1

Gene DB identifier	Gene Organism . Short Name	Homologues Homologue . Primary Identifier	Homologue Symbol	Homologue Description	Homologues Homologue . Organism . Short Name	Data Sets Name	Homologue Pathways
117230285	B. vosnesenskii	34614	CG6746	uncharacterized protein	D. melanogaster	HGD-Ortho data set	7 Pathways
117230393	B. vosnesenskii	326184	CG32052	uncharacterized protein	D. melanogaster	HGD-Ortho data set	0 Pathways
117230408	B. vosnesenskii	42656	AdipoR	adiponectin receptor	D. melanogaster	HGD-Ortho data set	0 Pathways
117230486	B. vosnesenskii	43186	CG33970	uncharacterized protein	D. melanogaster	HGD-Ortho data set	0 Pathways
117230518	B. vosnesenskii	41889	Rh6	rhodopsin 6	D. melanogaster	HGD-Ortho data set	7 Pathways
117230518	B. vosnesenskii	42261	Rh2	rhodopsin 2	D. melanogaster	HGD-Ortho data set	0 Pathways
117230518	B. vosnesenskii	42367	ninaE	neither inactivation nor afterpotential E	D. melanogaster	HGD-Ortho data set	0 Pathways
117230575	B. vosnesenskii	40145	Oat	ornithine aminotransferase precursor	D. melanogaster	HGD-Ortho data set	4 Pathways
117230690	B. vosnesenskii	40252	CG33969	uncharacterized protein	D. melanogaster	HGD-Ortho data set	7 Pathways
117230738	B. vosnesenskii	34135	Scgalpha	sarcoglycan alpha	D. melanogaster	HGD-Ortho data	0 Pathways

Figure S10. The result of making pathways optional allows additional homologues to appear in the table. The pathway information is now shown as embedded tables in the last column.

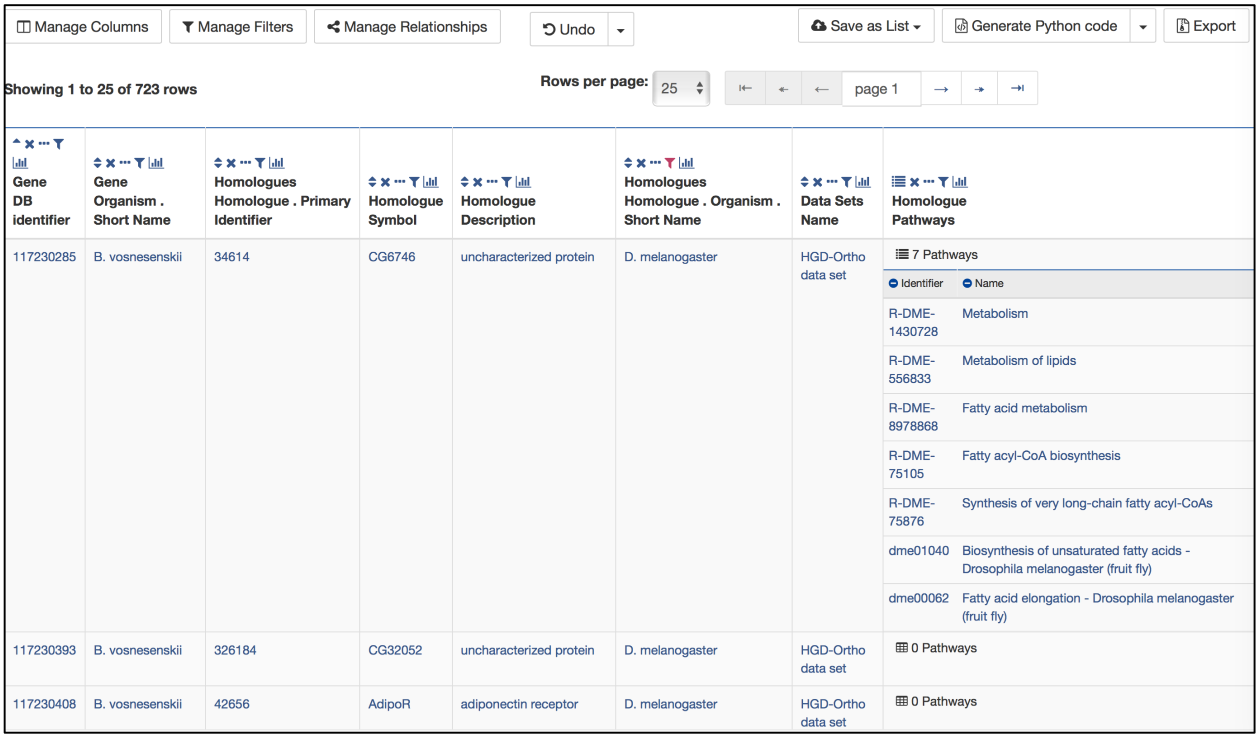

Manage Columns | Manage Filters | Manage Relationships | Undo | Save as List | Generate Python code | Export

Showing 1 to 25 of 723 rows

Rows per page: 25 | page 1

Gene DB identifier	Gene Organism . Short Name	Homologues Homologue . Primary Identifier	Homologue Symbol	Homologue Description	Homologues Homologue . Organism . Short Name	Data Sets Name	Homologue Pathways
117230285	B. vosnesenskii	34614	CG6746	uncharacterized protein	D. melanogaster	HGD-Ortho data set	7 Pathways
117230393	B. vosnesenskii	326184	CG32052	uncharacterized protein	D. melanogaster	HGD-Ortho data set	0 Pathways
117230408	B. vosnesenskii	42656	AdipoR	adiponectin receptor	D. melanogaster	HGD-Ortho data set	0 Pathways

7 Pathways (embedded table for 117230285):

Identifier	Name
R-DME-1430728	Metabolism
R-DME-556833	Metabolism of lipids
R-DME-8978868	Fatty acid metabolism
R-DME-75105	Fatty acyl-CoA biosynthesis
R-DME-75876	Synthesis of very long-chain fatty acyl-CoAs
dme01040	Biosynthesis of unsaturated fatty acids - Drosophila melanogaster (fruit fly)
dme00062	Fatty acid elongation - Drosophila melanogaster (fruit fly)

Figure S11. Expanding pathway information for a single homologue.

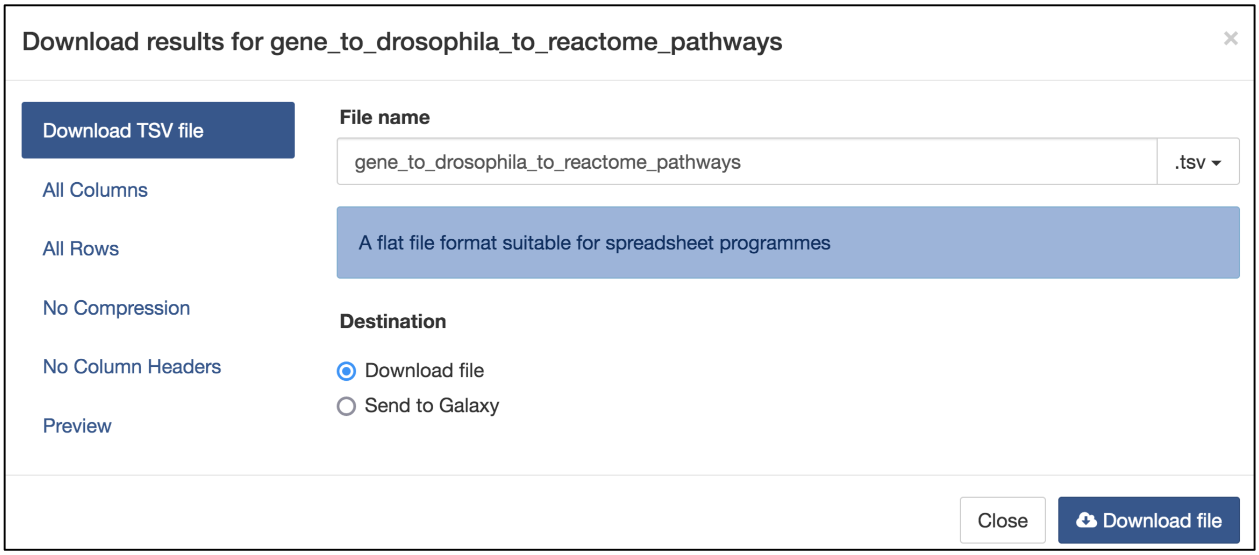

Figure S12. The Export menu. When the table shown in Figures S11 and S12 is exported, the table format will change so that pathway information is no longer embedded. The pathway cells for rows without pathways will contain missing values.

Part II. HymenopteraMine Application Programming Interface

Getting started

Step 1. The first step is to download the InterMine libraries. All InterMine API libraries (Java, Perl, Python, Ruby, JavaScript, R) are available here:

https://intermine.readthedocs.io/en/latest/web-services/#api-and-client-libraries

However, for this example we will use the Python libraries which can be installed with pip rather than the URL above.

We will use Python 3.8, and as of writing this example, for any release above Python 3.7 it is necessary to install the "dev" branch of InterMine as changes to make code compatible with Python 3.7+ have not yet been added to the latest InterMine release.

```
pip3 install https://github.com/intermine/intermine-ws-
python/archive/refs/heads/dev.zip
```

However, if you are using Python 2 or a Python 3 release earlier than 3.7, you can use the latest InterMine release using the appropriate version of pip (for Python 2 or 3):

```
pip install intermine
```

Next, install any Python dependencies if prompted by pip.

Step 2. Obtain an API key using the HymenopteraMine web interface.

Login through the web interface, click 'MyMine', then click the 'Account Details' tab. Find the 'API Access Key' section. If no API key exists, it will say 'No API key has been allocated to this account'. In that case, click the 'Generate a new API key' button to create one.

Once the key has been created (either in the above step or from a previous session), it will appear in the outlined box. Use this key for any examples involving interaction with your account (e.g., saving a list).

Examples

Example 1: Run a template query on a single Gene ID

This example shows how to run the template query 'Gene ID → D. melanogaster homologues and their Reactome pathways' on a single Gene ID.

Navigate to the HymenopteraMine homepage and scroll down to see the template category bar in the middle of the page (Figure S6 above). Look for the template named 'Gene ID → D. melanogaster homologues and their Reactome pathways' under 'Homology' and click it.

Each template has links at the bottom of the gray box that, when clicked, generate code to run that template in Perl, Python, Ruby, or Java (Figure S7 above). Click the "Python" link, and a new browser window will open with sample Python code for this template.

Create a Python script from this code by copying and pasting it into a text editor.

Next, choose a Gene ID for the template query. The script uses the default value for Gene.primaryIdentifier = 114870968. Edit this value to run the template query on a different Gene ID, for example, the *B. vosnesenskii* Gene ID = 117231166.

You may also modify the print statement to choose which column values to display (all columns are displayed by default).

```
#!/usr/bin/env python

# The line below will be needed if you are running this script with python 2.
# Python 3 will ignore it.
from __future__ import print_function

# The following two lines will be needed in every python script:
from intermine.webservice import Service
service = Service("http://128.206.116.35/hymenopteramine/service")

# Given a Gene ID, retrieve Drosophila melanogaster homologues and their
# Reactome pathways. A homologue will not be listed if it does not have a
# Reactome pathway. Depending on the species of the input gene, the results
# may include both HGD-Ortho and OrthoDB datasets, which can be filtered from
# the output table.

template = service.get_template('gene_to_drosophila_to_reactome_pathways')

# You can edit the constraint values below
# A    Gene.primaryIdentifier

rows = template.rows(
    A = {"op": "=", "value": "117231166"}
)
for row in rows:
    print(row["primaryIdentifier"], row["organism.shortName"], \
        row["homologues.homologue.primaryIdentifier"],
row["homologues.homologue.symbol"], \
        row["homologues.homologue.description"],
row["homologues.homologue.organism.shortName"], \
        row["homologues.dataSets.name"],
row["homologues.homologue.pathways.identifier"], \
        row["homologues.homologue.pathways.name"])
```

Output from running this script:

```
17231166 B. vosnesenskii 43253 RYa-R RYamide receptor D. melanogaster HGD-
Ortho data set R-DME-162582 Signal Transduction
117231166 B. vosnesenskii 43253 RYa-R RYamide receptor D. melanogaster HGD-
Ortho data set R-DME-372790 Signaling by GPCR
117231166 B. vosnesenskii 43253 RYa-R RYamide receptor D. melanogaster HGD-
Ortho data set R-DME-388396 GPCR downstream signalling
117231166 B. vosnesenskii 43253 RYa-R RYamide receptor D. melanogaster HGD-
Ortho data set R-DME-418555 G alpha (s) signalling events
```

To view all of the editable constraints for the template query, print the contents of `template.constraint_dict`. It is helpful to use interactive python via the command line to obtain these values:

```
>>> from intermine.webservice import Service
>>> service = Service("http://128.206.116.35/hymenopteramine/service")
>>> template=service.get_template('gene_to_drosophila_to_reactome_pathways')
>>> template.constraint_dict
{'A': <TemplateBinaryConstraint: Gene.primaryIdentifier = 114870968
(editable, locked)>, 'B': <TemplateBinaryConstraint:
Gene.homologues.homologue.organism.shortName = D. melanogaster (non-editable,
locked)>}
```

For the above template, Gene.primaryIdentifier is the only editable constraint.

Example 2: Run a template query on a list of Gene IDs

This example shows how to run the template query 'Gene ID → D. melanogaster homologues and their Reactome pathways' on a list of Gene IDs.

Begin by generating the Python code for this template as in Example 1. The code will be customized to run the query on a list instead of a single Gene ID. The steps performed in the script are explained below, and the final Python script is presented at the end.

Create a file named 'identifiers.txt' that contains a list of Gene IDs, one per line. In this example, the input file contains a list of 505 *B. vosnesenskii* Gene IDs (Supplementary File 2). These IDs are loaded into an array named 'identifiers'.

```
identifiers = []
with open("identifiers.txt") as f:
    for line in f:
        identifiers.append(line.rstrip())
```

Here are the Gene IDs used in this example:

```
>>> identifiers
['117230162', '117230199', '117230285', '117230291', '117230346',
'117230358', '117230362', '117230363', '117230393', '117230408', '117230486',
'117230511', '117230518', '117230575', '117230629', '117230634', '117230653',
'117230690', '117230738', '117230754', '117230781', '117230793', '117230799',
'117230807', '117230814', '117230820', '117230829', '117230889', '117230938',
'117231001', '117231004', '117231006', '117231020', '117231087', '117231146',
'117231158', '117231164', '117231166', '117231173', '117231179', '117231184',
'117231192', '117231198', '117231263', '117231279', '117231280', '117231333',
'117231338', '117231350', '117231366', '117231401', '117231457', '117231475',
'117231497', '117231570', '117231577', '117231588', '117231643', '117231663',
'117231690', '117231704', '117231708', '117231757', '117231773', '117231804',
'117231851', '117231866', '117231877', '117231884', '117231929', '117231970',
'117232009', '117232059', '117232072', '117232079', '117232091', '117232117',
'117232158', '117232160', '117232211', '117232285', '117232365', '117232476',
'117232485', '117232516', '117232520', '117232521', '117232543', '117232555',
'117232632', '117232638', '117232669', '117232722', '117232741', '117232759',
'117232781', '117232815', '117232820', '117232842', '117232854', '117232885',
'117232898', '117232991', '117232995', '117233106', '117233107', '117233119',
'117233138', '117233152', '117233180', '117233196', '117233202', '117233209',
```

```
'117233220', '117233279', '117233298', '117233304', '117233335', '117233346',
'117233347', '117233379', '117233409', '117233410', '117233507', '117233510',
'117233515', '117233535', '117233548', '117233562', '117233573', '117233575',
'117233578', '117233622', '117233634', '117233669', '117233673', '117233698',
'117233700', '117233728', '117233782', '117233814', '117233860', '117233884',
'117233992', '117234004', '117234007', '117234012', '117234046', '117234072',
'117234074', '117234076', '117234117', '117234154', '117234159', '117234169',
'117234188', '117234238', '117234252', '117234257', '117234267', '117234276',
'117234283', '117234285', '117234352', '117234401', '117234416', '117234443',
'117234459', '117234485', '117234486', '117234547', '117234553', '117234570',
'117234573', '117234694', '117234799', '117234804', '117234818', '117234841',
'117235005', '117235006', '117235040', '117235044', '117235050', '117235056',
'117235074', '117235086', '117235126', '117235235', '117235244', '117235255',
'117235288', '117235293', '117235301', '117235332', '117235357', '117235409',
'117235447', '117235449', '117235455', '117235493', '117235588', '117235675',
'117235702', '117235722', '117235777', '117235780', '117235904', '117235946',
'117235949', '117236002', '117236024', '117236040', '117236091', '117236095',
'117236101', '117236103', '117236165', '117236187', '117236192', '117236198',
'117236222', '117236245', '117236269', '117236270', '117236343', '117236344',
'117236365', '117236366', '117236367', '117236374', '117236376', '117236395',
'117236403', '117236405', '117236431', '117236441', '117236463', '117236473',
'117236485', '117236506', '117236511', '117236521', '117236522', '117236533',
'117236539', '117236580', '117236625', '117236641', '117236689', '117236707',
'117236709', '117236721', '117236722', '117236768', '117236801', '117236828',
'117236829', '117236830', '117236850', '117236856', '117236910', '117236934',
'117236940', '117236942', '117236957', '117236972', '117236973', '117236974',
'117236977', '117237037', '117237057', '117237096', '117237098', '117237232',
'117237252', '117237253', '117237257', '117237293', '117237339', '117237409',
'117237410', '117237464', '117237494', '117237523', '117237627', '117237681',
'117237721', '117237723', '117237747', '117237787', '117237834', '117237847',
'117237930', '117237977', '117237998', '117238022', '117238023', '117238026',
'117238029', '117238063', '117238072', '117238142', '117238150', '117238171',
'117238194', '117238253', '117238294', '117238297', '117238301', '117238314',
'117238315', '117238329', '117238394', '117238404', '117238471', '117238490',
'117238562', '117238564', '117238578', '117238584', '117238653', '117238669',
'117238678', '117238722', '117238732', '117238744', '117238802', '117238926',
'117238959', '117238961', '117238983', '117239073', '117239095', '117239096',
'117239098', '117239118', '117239127', '117239133', '117239136', '117239141',
'117239152', '117239158', '117239162', '117239213', '117239215', '117239274',
'117239413', '117239503', '117239537', '117239565', '117239572', '117239575',
'117239576', '117239577', '117239578', '117239624', '117239684', '117239715',
'117239716', '117239719', '117239794', '117239870', '117240005', '117240015',
'117240024', '117240035', '117240039', '117240058', '117240059', '117240061',
'117240066', '117240087', '117240169', '117240182', '117240189', '117240206',
'117240225', '117240275', '117240280', '117240281', '117240337', '117240347',
'117240348', '117240350', '117240352', '117240375', '117240393', '117240411',
'117240417', '117240583', '117240630', '117240649', '117240655', '117240943',
'117240949', '117240952', '117240985', '117241024', '117241241', '117241653',
'117241728', '117241744', '117241791', '117241816', '117241823', '117241831',
'117241832', '117241844', '117241845', '117241846', '117241885', '117241890',
'117241908', '117241912', '117242007', '117242052', '117242057', '117242067',
'117242075', '117242079', '117242096', '117242101', '117242115', '117242122',
'117242185', '117242244', '117242277', '117242319', '117242347', '117242354',
'117242381', '117242400', '117242488', '117242565', '117242567', '117242599',
'117242641', '117242677', '117242759', '117242767', '117242790', '117242792',
'117242800', '117242802', '117242819', '117242822', '117242824', '117242830',
'117242846', '117242876', '117242884', '117242945', '117242970', '117242988',
```

```
'117242995', '117243022', '117243038', '117243073', '117243093', '117243094',
'117243104', '117243116', '117243136', '117243137', '117243138', '117243139',
'117243141', '117243142', '117243143', '117243148', '117243154', '117243202',
'117243295', '117243296', '117243318', '117243322', '117243323', '117243324',
'117243329', '117243342', '117243345', '117243347', '117243354', '117243360',
'117243373', '117243378', '117243423', '117243426', '117243443', '117243480',
'117243492', '117243497', '117243504', '117243505', '117243518', '117243521',
'117243524', '117243538', '117243556', '117243596', '117243631', '117243647',
'117243649', '117243650']
```

Next, a list named 'My Example B. vosnesenskii list' will be created and save to your HymenopteraMine account.

To interact with your account, the service line at the top of the script must be modified to include your API key. Add a second parameter of the form 'token = "YOUR-API-KEY", replacing YOUR-API-KEY with the API key from 'Account Details' under the 'MyMine' tab, as previously obtained in Step 2 above.

```
from intermine.webservice import Service
service = Service("http://128.206.116.35/hymenopteramine/service", token =
"YOUR-API-KEY")
lm=service.list_manager()
lm.create_list(content=identifiers,list_type="Gene",name="My Example B.
vosnesenskii list")
```

Then the template query will run on the Gene IDs listed in identifiers.txt, and all columns of the resulting table will be printed.

```
template = service.get_template('gene_to_drosophila_to_reactome_pathways')
rows = template.rows(A = {"op": "IN", "value": "My Example B. vosnesenskii
list"})
for row in rows:
    print(row["primaryIdentifier"], row["organism.shortName"], \
        row["homologues.homologue.primaryIdentifier"],
row["homologues.homologue.symbol"], \
        row["homologues.homologue.description"],
row["homologues.homologue.organism.shortName"], \
        row["homologues.dataSets.name"],
row["homologues.homologue.pathways.identifier"], \
        row["homologues.homologue.pathways.name"])
```

Here are the first five lines of output:

```
117230285 B. vosnesenskii 34614 CG6746 uncharacterized protein D.
melanogaster HGD-Ortho data set R-DME-1430728 Metabolism
117230285 B. vosnesenskii 34614 CG6746 uncharacterized protein D.
melanogaster HGD-Ortho data set R-DME-556833 Metabolism of lipids
117230285 B. vosnesenskii 34614 CG6746 uncharacterized protein D.
melanogaster HGD-Ortho data set R-DME-75105 Fatty acyl-CoA biosynthesis
117230285 B. vosnesenskii 34614 CG6746 uncharacterized protein D.
melanogaster HGD-Ortho data set R-DME-75876 Synthesis of very long-chain
fatty acyl-CoAs
117230285 B. vosnesenskii 34614 CG6746 uncharacterized protein D.
melanogaster HGD-Ortho data set R-DME-8978868 Fatty acid metabolism
```

There are 6,635 lines total:

```
>>> len(rows)
6635
```

As in the previous example, the print statement may be altered to print only the desired columns. Note that once a list is created, it is saved to your account and cannot be recreated with the same name. To access an existing list, use `get_list()`.

```
lm=service.list_manager()
l1=lm.get_list(name="My Example B. vosnesenskii list")
```

Iterate over the list object to print the items in the list:

```
for row in l1:
    print(row)
```

Delete lists by passing an array of list names to delete_lists().

```
lm.delete_lists(["My Example B. vosnesenskii list"])
```

Here is the standalone Python script for this example:

```
#!/usr/bin/env python3

# The line below will be needed if you are running this script with python 2.
# Python 3 will ignore it.
from __future__ import print_function

# The following two lines will be needed in every python script:
from intermine.webservice import Service
service = Service("http://128.206.116.35/hymenopteramine/service", token =
"YOUR-API-KEY")

identifiers = []
with open("identifiers.txt") as f:
    for line in f:
        identifiers.append(line.rstrip())

# Create and save list of Gene IDs:
lm=service.list_manager()
lm.create_list(content=identifiers,list_type="Gene",name="My Example B.
vosnesenskii list")

# Given a Gene ID, retrieve Drosophila melanogaster homologues and their
# Reactome pathways. A homologue will not be listed if it does not have a
# Reactome pathway. Depending on the species of the input gene, the results
# may include both HGD-Ortho and OrthoDB datasets, which can be filtered from
# the output table.

template = service.get_template('gene_to_drosophila_to_reactome_pathways')
rows = template.rows(A = {"op": "IN", "value": "My Example B. vosnesenskii
list"})
for row in rows:
    print(row["primaryIdentifier"], row["organism.shortName"], \
        row["homologues.homologue.primaryIdentifier"],
row["homologues.homologue.symbol"], \
        row["homologues.homologue.description"],
row["homologues.homologue.organism.shortName"], \
        row["homologues.dataSets.name"],
row["homologues.homologue.pathways.identifier"], \
        row["homologues.homologue.pathways.name"])
```
